# A Robust Heparin‐Mimicking Polyglycerol‐Based Coating for Blood‐Contacting Devices with Long‐Term Hemocompatibility and Preliminary Anti‐Inflammatory Properties

**DOI:** 10.1002/adhm.202502766

**Published:** 2025-09-21

**Authors:** Kunpeng Liu, Philip Nickl, Jun Feng, Rainer Haag

**Affiliations:** ^1^ Institute for Chemistry and Biochemistry Freie Universität Berlin Takustr. 3 14195 Berlin Germany; ^2^ INM‐Leibniz Institute for New Materials Campus D2 2 66123 Saarbrücken Germany

**Keywords:** anti‐inflammatory, Blood‐contact materials and devices, surface modification, long‐term anti‐thrombosis property, polyglycerols

## Abstract

Blood‐contacting medical devices play a crucial role in clinical interventions, but their susceptibility to thrombosis and inflammation poses serious risks to treatment outcomes and patient safety. This study presents a novel coating that combines dendritic polyglycerol amine (dPGA), dendritic polyglycerol aldehyde (dPG‐CHO), and linear polyglycerol sulfate (lPGS) using a layer‐by‐layer self‐assembly method (LBL) on a polystyrene surface. The immobilization of dendritic polyglycerol enhances surface coverage, enabling the incorporation of a higher density of heparin‐mimicking lPGS, while the covalent bonding ensures the coating's long‐term stability. Compared to the pristine substrate, the coating significantly reduced platelet adhesion and activation. Notably, its hemocompatibility effects persist even after 30 days. Furthermore, co‐incubation experiments with RAW264.7 macrophages confirmed the anti‐inflammatory properties of the polyglycerol‐based coating. These results demonstrate that this heparin‐mimetic coating effectively improves the hemocompatibility of polystyrene and has the potential to be applied to other blood‐contacting materials.

## Graphical abstract

A robust heparin‐mimicking coating based on sulfated polyglycerol is constructed via layer‐by‐layer assembly on bloodcontacting surfaces. The coating exhibits long‐term anticoagulant and anti‐inflammatory properties by resisting protein adsorption, preventing platelet activation, and suppressing immune responses, offering a promising strategy for improving the hemocompatibility and durability of cardiovascular devices.

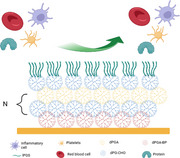

## 1 Introduction

Advanced stages of cardiovascular disease typically require the use of implantable medical devices.^[^ ^1^ ^]^ However, these devices face several complications, with thrombosis and inflammation being particularly challenging. These adverse reactions, in addition to causing device failure, can require patients to take medications for the rest of their lives. For instance, anti‐coagulants like heparin and vitamin K antagonists have seen broad clinical use in preventing thrombosis for patients with cardiovascular implants. However, such blood‐thinning drugs can also increase the risk of potentially life‐threatening bleeding.^[^ ^2^ ^]^ In the face of these challenges, improving the biocompatibility of the implantable devices and materials is a promising strategy to reduce complications. The medical device industry has traditionally prioritized mechanical properties—such as strength, fatigue resistance, and flexibility—during fabrication. As a result, surface properties, which play a critical role in triggering biological responses, have often been overlooked.

Surface modification is a widely used and effective strategy to improve the biocompatibility of a material's surface without changing the material's bulk properties, including physical adsorption, covalent bonding, and biological methods.^[^ ^3^ ^]^ Numerous studies have investigated different surface coatings to resist the adhesion of blood proteins and platelets. Two widely used surface modification strategies are the grafting of functional molecules and the application of hydrogel coatings.^[^ ^3^, ^4^ ^]^ For molecular grafting, hydrophilic polymers are most commonly employed. Polyethylene glycol (PEG), recognized as the “gold standard” biocompatible polymer, is frequently used for modifying blood‐contacting devices. As an FDA‐approved material, PEG has been extensively applied in clinical settings. However, its widespread use has led to an increasing number of patients developing anti‐PEG antibodies, which can reduce its effectiveness and even trigger hypersensitivity reactions and other adverse immune responses.^[^ ^5^ ^]^ Furthermore, for polymer coatings, one major challenge is achieving complete and uniform surface coverage with thin layers.^[^ ^6^ ^]^ There are concerns about the long‐term durability and limited effectiveness of these coatings under prolonged use.^[^ ^7^ ^]^ To address these issues, hydrogel coatings have emerged as a promising alternative, such as PEG‐based hydrogel,^[^ ^8^ ^]^ zwitterionic polymer‐based hydrogel,^[^ ^9^ ^]^ and so on. However, a key limitation of hydrogel coatings is the difficulty in controlling their thickness, which can range from tens to hundreds of micrometers.^[^ ^10^ ^]^ Excessive coating thickness may compromise the performance of medical devices such as vascular grafts and blood‐transfer catheters. Furthermore, mismatches between the bulk properties of the coating and the substrate can lead to mechanical instability. Thick hydrogel layers tend to swell, which may result in delamination or detachment from the surface. Detached fragments can even circulate in the bloodstream, posing a risk of vascular occlusion.^[^ ^11^ ^]^ Therefore, there remains an urgent need for stable coatings with controllable and appropriate thickness to ensure both the biocompatibility and the mechanical integrity of blood‐contacting medical devices.

Layer‐by‐layer self‐assembly is an effective technique for fabricating polyelectrolyte coatings by sequentially depositing oppositely charged species, allowing for the precise construction of biofunctional surfaces.^[^ ^12^ ^]^ However, maintaining the stability of these coatings under environmental stress remains a significant challenge.^[^ ^13^ ^]^ Heparin, a widely used anticoagulant, is effective at preventing thrombosis but also carries a considerable risk of bleeding.^[^ ^14^ ^]^ Although some studies have incorporated heparin into LBL coatings to enhance the anticoagulant properties of vascular grafts, ensuring such coatings’ stability and sustained bioactivity remains a key concern.^[^ ^15^ ^]^ As an alternative, synthetic polyelectrolytes that mimic the biological functions of heparin and heparan sulfate offer potential advantages by mitigating heparin's inherent risks.^[^ ^1^, ^16^ ^]^ Among these, polyglycerol sulfate has emerged as a particularly promising candidate due to its anticoagulant and anti‐inflammatory properties. Over the past decade, it has been extensively studied in solution‐based systems, demonstrating strong performance in both in‐vitro and in‐vivo models.^[^ ^17^ ^]^ Nevertheless, research involving polyglycerol sulfate‐coated surfaces is still sparse, underlining the need for further research to improve such coatings’ stability and functional performance.

**Scheme 1:**
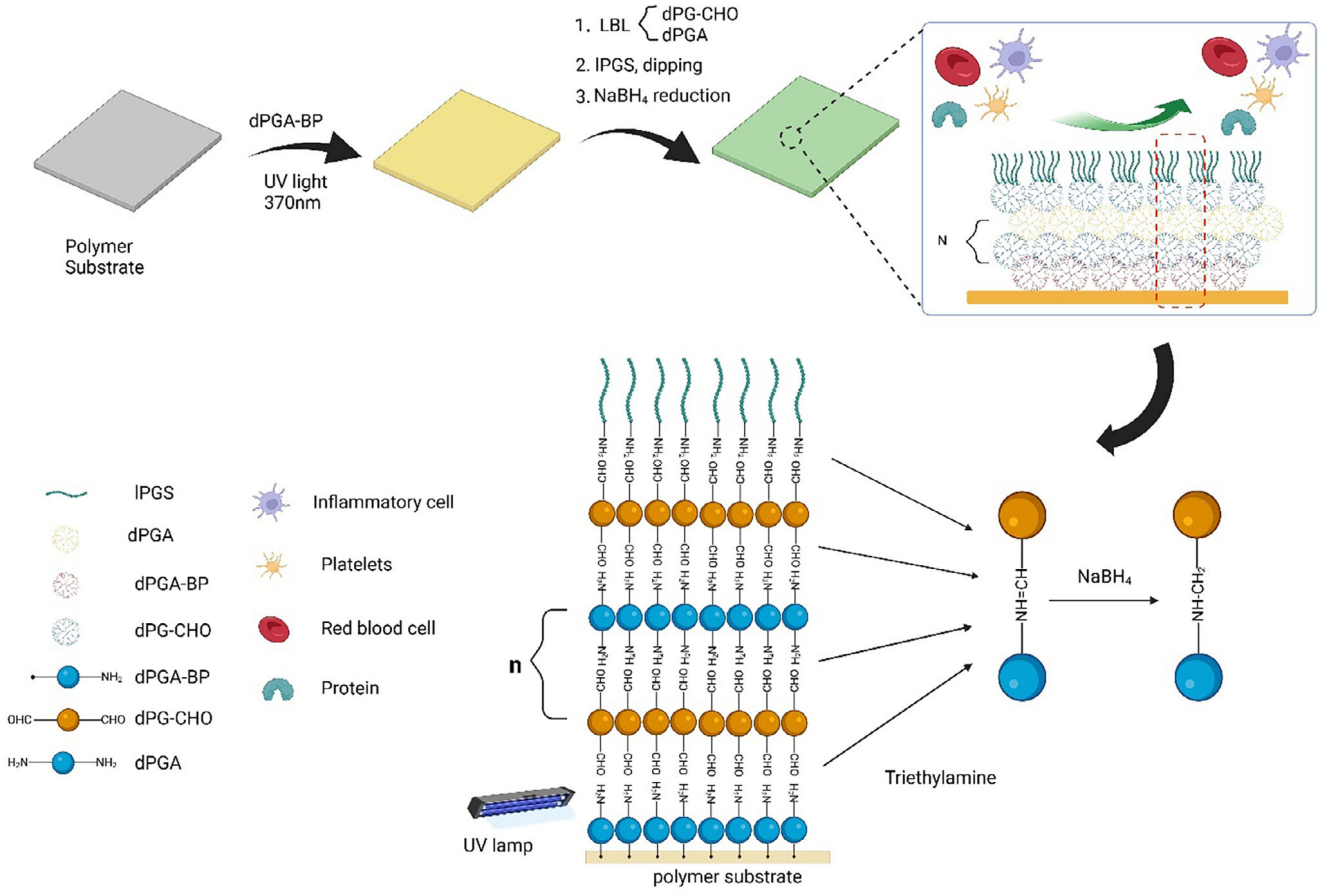
Schematic illustrations of coating, created in BioRender. Schematic illustration of the polyglycerol‐based coating process. dPGA‐BP is photo‐crosslinked onto a polymer substrate under UV light (370 nm), followed by layer‐by‐layer assembly of dPG‐CHO and dPGA via reversible Schiff base bonds. lPGS was grafed on the top finally.Then the imine bonds were reduced to stable amine.

Based on the above considerations, we developed a covalently fixed polyglycerol‐based coating to functionalize polystyrene surfaces using a distinct combination of aldehyde‐functionalized dendritic polyglycerol, amine‐functionalized polyglycerol, and benzophenone‐modified dPGA (dPGA‐BP), together with sulfated linear polyglycerol. The overall design is illustrated in **Scheme** **1**. A modified dendritic polyglycerol containing a benzophenone moiety was employed as the anchoring molecule, allowing covalent attachment to the polystyrene backbone under UV activation. Meanwhile, the terminal amino groups (‐NH_2_) served as reactive sites for the introduction of additional macromolecules. The polyelectrolyte lPGS, which structurally mimics heparin, was incorporated to resist platelet activation and adhesion. Additionally, lPGS acts as a competitive inhibitor to reduce inflammatory responses. Unlike traditional heparin‐like or polyionic coatings, our approach produced a hydrolysis‐resistant and highly negatively charged network simultaneously with improved long‐term stability under physiological conditions. Specifically, by combining chemical bonding with an LBL approach, we achieved a higher grafting density and broader surface coverage. Furthermore, the imine bonds initially formed between dPGA and dPG‐CHO were reduced to more stable amine linkages, enhancing the coating's long‐term stability. To evaluate the anti‐inflammatory and prolonged hemocompatibility performance of the coating, in‐vitro platelet adhesion assays were conducted at various time points, along with co‐incubation experiments using RAW264.7 macrophages. Surface characterization and cytocompatibility assessments were also performed. With these comprehensive results, we expected to demonstrate a simple yet promising surface modification strategy for improving the long‐term antithrombotic and anti‐inflammatory performance of blood‐contacting materials.

## 2 Results and Discussion

### 2.1 Polymer Synthesis

#### 2.1.1 Synthesis of Amino‐Modified lPGS

In this study, a block copolymer was synthesized via anionic ring‐opening polymerization, as illustrated in Figure S1 (Supporting Information). Anionic polymerization is a type of living polymerization that allows precise control over polymer architecture, making it particularly suitable for synthesizing block copolymers. In our approach, ethixyethyl glycidyl ether (EEGE) was polymerized first, followed by the addition of allyl glycidyl ether (AGE) to form the block copolymer, referred to as lPG‐b‐20AGE. The molecular weight of the block copolymer was ascertained by gel permeation chromatography (GPC) (Figure S2, Supporting Information), yielding a weight‐average molecular weight (Mw) of 10 566 g mol^−1^, a number‐average molecular weight (Mn) of 8033 g mol^−1^, and a polydispersity index (PDI) of 1.31. The structure of the polymer was characterized by proton nuclear magnetic resonance spectroscopy (^1^H NMR) ^1^, as shown in Figure S3 (Supporting Information). Peaks at around 5.90 and 5.20 ppm corresponded to protons on the double bonds, while peaks in the range of 3.40 – 3.80 ppm are attributed to protons in the polymer backbone. After polymerization, the block copolymer was sulfated. Following dialysis and lyophilization, the polymer was grafted with cysteamine to introduce amino groups, serving as linker sites for surface attachment. The final polymer was then analyzed by ^1^H‐NMR (Figure S4, Supporting Information). A downfield shift of the proton signals adjacent to the ‐SO_3_^‐^ groups to the range of 4.30 – 3.90 ppm confirmed the successful synthesis of the lPGS copolymer. At the same time, 4 peaks from 3.20 to 1.80 ppm confirmed that the cysteamine was grafted successfully. In summary, these results demonstrated that the lPGS was successfully synthesized and functionalized.

#### 2.1.2 Synthesis of BP Modified of dPGA

To enable anchoring of the molecule onto the polystyrene surface, a benzophenone moiety was introduced into the system. Specifically, 4‐benzoylbenzoic acid was conjugated to dPGA via a dehydration condensation reaction between the amine and carboxylic acid groups. The resulting product was characterized by Fourier‐transform infrared spectroscopy (FTIR). As shown in Figure S5 (Supporting Information), the ‐C─H stretching vibration band near 3000 cm^−1^ became more pronounced after the grafting of benzophenone, likely due to the enhanced stretching vibrations induced by the aromatic ring. Additionally, compared to the spectrum of dPGA, a new absorption band at 1650 cm^−1^ appeared in the spectrum of dPGA‐BP, corresponding to the C═O stretching vibration of the benzophenone unit. Another distinct peak appeared at 1535 cm^−1^, which is assigned to the stretching vibration of the aromatic ring. These spectral changes collectively confirm the successful grafting of the benzophenone moiety onto the dPGA structure.

#### 2.1.3 Synthesis of dPG‐CHO

To enable conjugation with dPGA via Schiff base formation, dPG‐CHO was synthesized by oxidizing all o‐hydroxyl groups on polyglycerol (dPG) to aldehyde groups. The resulting product was characterized using FTIR. As shown in Figure S6 (Supporting Information), in contrast to unmodified dPG, a distinct absorption peak appeared at 1728 cm^−1^, corresponding to the stretching vibration of the aldehyde (‐CHO) group. This result confirmed the successful synthesis of dPG‐CHO.

### 2.2 Coating Characterization

Different polymers were grafted onto the polystyrene (PS) surface via a covalent LBL assembly strategy specifically designed to overcome the instability of electrostatic assemblies. First, dPGA‐BP was immobilized on the PS substrate, introducing amine functional groups on the surface. Upon UV irradiation, the benzophenone moiety underwent photolysis, forming a reactive triplet‐state ketone. The resulting excited state enabled covalent bond formation by inserting into C─H bonds and other functional groups on the surface.^[^ ^18^ ^]^ Then, alternating layers of dPG‐CHO and dPGA were sequentially deposited to form multilayers, with 1, 5, and 10 deposition cycles tested. The polymers were linked via Schiff base reactions. Finally, lPGS was grafted as the outermost layer. To further stabilize the coating, the imine (‐C═N) bonds were reduced to more stable amine groups by immersing the coated samples in a sodium borohydride (NaBH_4_) solution. This chemical stabilization substantially enhances coating durability in physiological environments, where purely electrostatic coatings often degrade. This reduction step improves chemical durability, as amine bonds are more stable due to their strong C–N single bonds and localized lone pairs, whereas imines are more reactive and prone to hydrolysis under physiological conditions. A sample naming convention was established based on two variables: the concentration of dPGA‐BP and the number of intermediate layers. For instance, PS‐2dPG‐lPGS, PS‐5dPG‐lPGS, and PS‐10dPG‐lPGS refer to coatings prepared using 2, 5, and 10 mg mL^−1^ of dPGA‐BP, respectively. In terms of layering, names like PS‐2dPG‐lPGS monolayer (1‐layer), 5‐layer, and 10‐layer indicate the number of dPG‐CHO/dPGA bilayers formed. A monolayer specifically refers to one complete cycle of dPG‐CHO and dPGA deposition.

**Figure 1:**
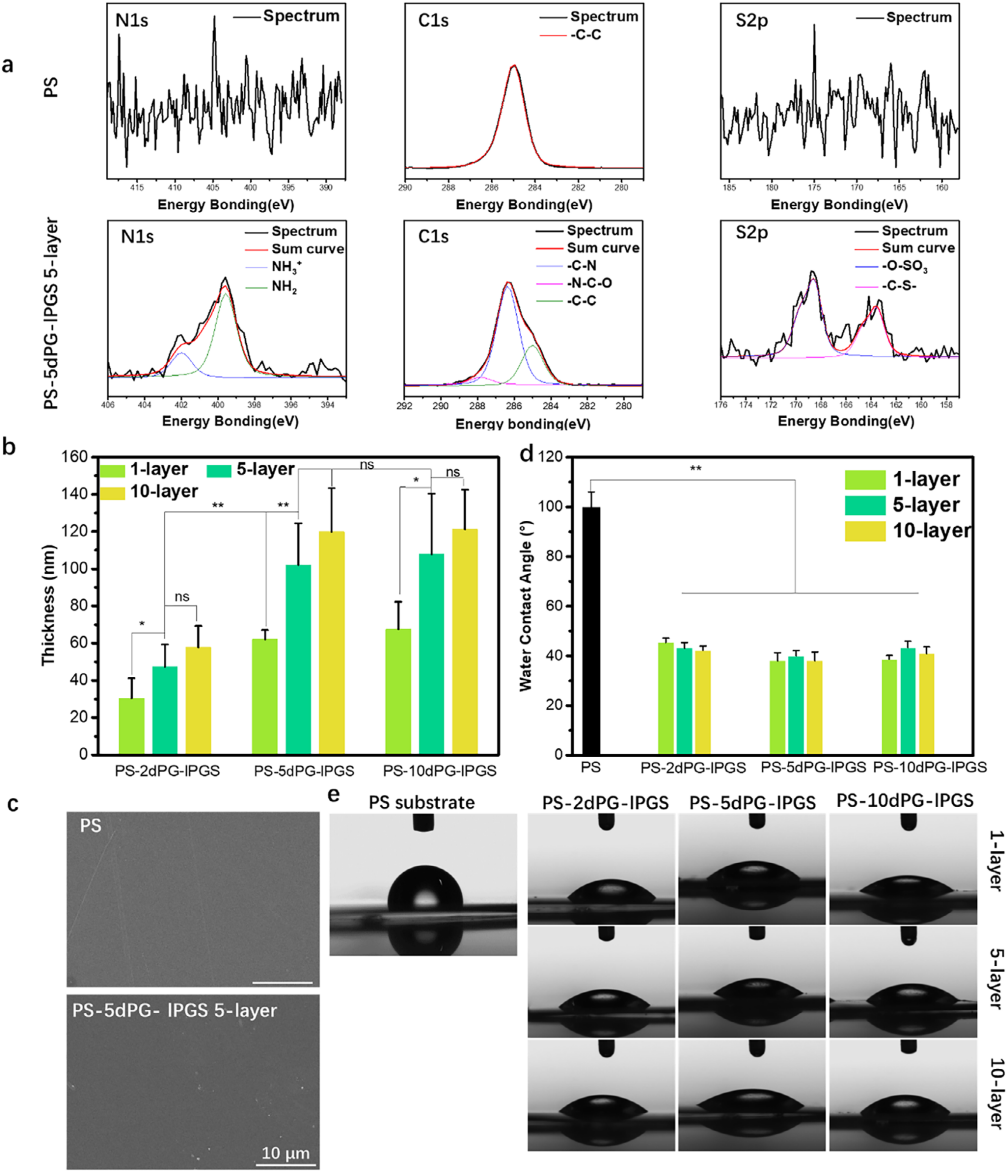
a) Normalized highly‐resolved elemental XP spectra of polystyrene and the coating PS‐5dPG‐lPGS 5‐layer. b) The thickness results of different coatings. c) SEM images of the surface of polystyrene and the coating PS‐5dPG‐lPGS 5‐layer. d) Water contact angles of different samples. e) Digital images of water contact angles of different samples. ^*^ *p*<0.05, ^**^ *p*<0.01.

This LBL approach offers two major advantages: 1) enhanced macromolecular loading on the PS surface, resulting in greater functional coverage, and 2) strong chemical grafting that ensures coating stability. Following surface modification, the coatings were characterized in terms of morphology, elemental composition, and wettability using scanning electron microscopy (SEM), X‐ray photoelectron spectroscopy (XPS), and water contact angle (WCA) measurements. The highly‐resolved elemental XP spectra (**Figure** **1**a) showed that nitrogen (N) and sulfur (S) were absent on the pristine PS surface. After coating, a strong signal appeared in the N 1s spectrum at ≈ 399.0 eV. This peak was deconvoluted into two components at binding energies of 399.5 and 402.0 eV, which were assigned to ‐NH_2_ groups and protonated amine groups (‐NH_3_^+^), respectively. The simultaneous presence of both ‐NH_2_ and ‐NH_3_^+^ species can be attributed to the use of a humidified (water‐containing) environment during sample preparation and analysis. Under such conditions, residual surface‐bound water molecules and local acidity may facilitate partial protonation of surface amine groups, resulting in a co‐existence of neutral and protonated forms. This effect is particularly likely given that the modified surface possesses a high surface charge, which enhances the affinity for water molecules and contributes to the formation of localized acidic microenvironments. The highly‐resolved C 1s spectrum also contained multiple components, including ‐C─C (285.0 eV), ‐C─N (286.4 eV), and ‐N─C─O (288.0 eV) bonds. In contrast, only a single carbon peak component for ‐C─C bonds was observed on the unmodified PS. For the highly‐resolved sulphur (S) S2p spectrum, no signal was present for the pristine PS, but distinct peaks were observed in the S2p spectrum after coating, originating from the lPGS layer. The main S 2p signal appeared ≈ 168.6 eV, corresponding to oxidized sulfur species such as sulfated groups (‐SO_3_^‐^), which are characteristic of the intended surface modification. A minor peak was also observed near 163.6 eV, which is not expected from the target structure and is likely due to trace sulfur‐containing impurities, such as residual thiol‐ or sulfide‐based reagents or intermediates from the synthesis process. These results confirm the successful incorporation of N and S elements and the formation of diverse chemical bonds on the modified surface.

To quantitatively determine the surface sulfonic acid group density, a Toluidine Blue O (TBO) dye‐binding assay was performed, and the results are shown in Figure S7 (Supporting Information). For the PS–2dPG–lPGS group, the ‐SO_3_^‐^ grafting densities for 1‐layer, 5‐layer, and 10‐layer coatings were 4.91 ± 2.25 nmol cm^−^^2^, 6.48 ± 2.72 nmol cm^−^^2^, and 11.23 ± 1.99 nmol cm^−^^2^, respectively. For the PS–5dPG–lPGS group, the corresponding values were 18.73 ± 4.60 nmol cm^−^^2^, 27.27 ± 3.24 nmol cm^−^^2^, and 30.77 ± 11.06 nmol cm^−^^2^, and for the PS–10dPG–lPGS group, the densities were 15.60 ± 4.12 nmol cm^−^^2^, 39.90 ± 11.03 nmol cm^−^^2^, and 35.38 ± 6.00 nmol cm^−^^2^, respectively. These results complemented the qualitative XPS data. Increasing the number of lPGS layers generally enhanced sulfonation, though a non‐linear trend at higher layers suggests possible saturation or shielding effects. Differences between groups likely arise from variations in available aldehyde sites for lPGS immobilization. Functionally, higher ‐SO_3_^‐^ densities increase surface negative charge, which may improve hemocompatibility by reducing protein adsorption and platelet adhesion. Due to surface irregularities of the PS sheet, direct measurement of coating thickness was challenging. Therefore, a flat, reflective silicon wafer was coated with a PS layer to serve as a uniform substrate for ellipsometry. The results (Figure 1b) showed that in the PS‐2dPG‐lPGS group, the coating thickness increased from 30.38 ± 10.80 nm (monolayer) to 47.46 ± 11.83 nm (5‐layer) and 57.77 ± 11.48 nm (10‐layer). For PS‐5dPG‐lPGS, thicknesses were 62.13 ± 4.94 nm (monolayer), 102.04 ± 22.40 nm (5‐layer), and 119.95 ± 23.36 nm (10‐layer). Similarly, the PS‐10dPG‐lPGS group showed values of 67.55 ± 14.67 nm, 107.86 ± 32.55 nm, and 121.20 ± 21.33 nm for the respective coating variations. These results indicate a consistent trend of increasing thickness with more layers, though the difference between 5‐layer and 10‐layer coatings was relatively small. Besides, to further assess coating uniformity, atomic force microscopy (AFM) was performed. For each sample, height images were acquired in tapping mode over a 10 µm × 10 µm scan area at three randomly selected regions, and the arithmetic mean roughness (Ra) was calculated from the flattened images. As shown in Figure S8 (Supporting Information), the Ra of unmodified PS was 0.19 ± 0.03 nm, while the PS‐5dPG‐lPGS 5‐layer coating exhibited an Ra of 1.08 ± 0.58 nm. These low and comparable values indicate a smooth and homogeneous surface. The surface morphology was further examined by SEM (Figure 1c). The unmodified PS and the PS‐5dPG‐lPGS 5‐layer coating both appeared smooth and homogeneous, with no apparent differences. Similar results were observed for other coating groups (see Figure S9, Supporting Information), confirming uniform coating morphology across all samples.

Wettability is a crucial factor for blood‐contacting materials. In general, hydrophilic surfaces show better compatibility with blood components, while hydrophobic surfaces tend to provoke adverse interactions. The water contact angle of different samples was measured to assess surface wettability, and the results are shown in Figure 1d,e. The unmodified PS surface exhibited a high WCA of 99.73 ± 6.35°, indicating strong hydrophobicity. However, after grafting with different polymers—namely dPGA, dPG‐CHO, and lPGS—the surface properties changed markedly, leading to a significant reduction in WCA. For the PS‐2dPG‐lPGS group, the WCA values for 1‐layer, 5‐layer and 10‐layer coatings were 45.40 ± 1.70°, 43.13 ± 2.26°, and 42.23 ± 1.75°, respectively. In the PS‐5dPG‐lPGS group, the corresponding WCA values were 38.75 ± 3.29°, 40.08 ± 2.12°, and 38.10 ± 3.47°. respectively. For the PS‐10dPG‐lPGS group, the WCA values were 38.56 ± 1.23°, 43.28 ± 2.64°, and 40.98 ± 2.67°, for the 1‐layer, 5‐layer, and 10‐layer coatings, respectively. These results demonstrate that the introduction of the polymer coatings significantly increased the hydrophilicity of the PS surface. This enhancement in wettability is attributed to the presence of the polyglycerol backbone and various hydrophilic functional groups such as ‐OH, ‐NH_2_, and ‐SO_3_^‐^ on the surface.

In summary, the observed changes in surface elemental composition, coating thickness, and wettability collectively confirm the successful construction of the functional coating on the PS surface.

### 2.3 Hemocompatibility Evaluation

**Figure 2:**
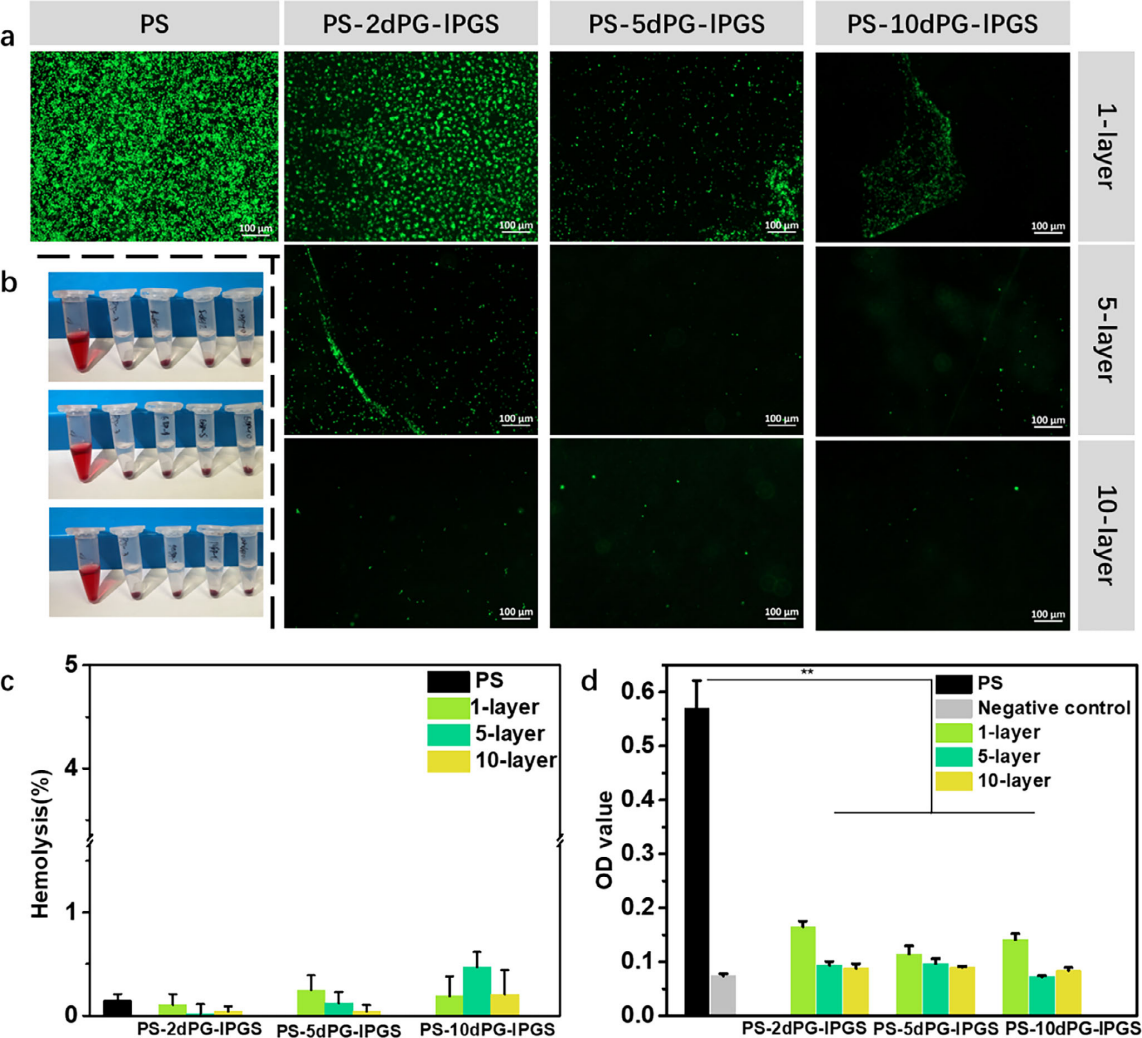
a) Fluoresence microscopy images of different samples after incubating platelets at 37 °C for 1 h. b) Digital images of hemolysis results. Images, from top to bottom: PS‐2dPG‐lPGS group, PS‐5dPG‐lPGS group, PS‐10dPG‐lPGS group. Samples, from left to right: disstilled water, PS. Monolayer, 5‐layer, 10‐layer coating. c) Hemolysis of different samples. d) LDH results of different samples after anti‐platelet‐adhesion experiment. ^*^ *p*<0.05, ^**^ *p*<0.01.

When foreign materials or implantable devices come into contact with flowing blood, a series of adverse biological reactions can occur. This process typically begins with the adsorption of plasma proteins onto the material surface, followed by platelet recognition, adhesion and activation. Once activated, platelets release various pro‐thrombotic factors, eventually leading to thrombosis formation. Therefore, the evaluation of surface platelet‐resistance properties is essential for assessing the hemocompatibility of blood‐contacting materials. To assess platelet adhesion and activation, platelet‐rich plasma (PRP) was used in the experiments. After co‐incubation of the materials with PRP, adherent platelets were stained with calcein AM, which fluoresces green, allowing for visualization. The results are shown in **Figure** **2**a, where the fluorescence images provide a comparative overview of platelet adhesion across different surface modifications.

On the unmodified PS surface, a strong green fluorescence signal was observed, with numerous distinct green spots and aggregates, indicating substantial platelet adhesion. In contrast, the monolayer‐coated groups showed varying degrees of improvement. For instance, although PS‐2dPG‐lPGS (1‐layer) exhibited reduced platelet adhesion compared to unmodified PS, a considerable portion of the surface remained fluorescent, suggesting persistent platelet attachment. PS‐5dPG‐lPGS (1‐layer) displayed a cleaner surface, though scattered green spots and occasional platelet aggregates were still visible. Among the monolayer samples, PS‐10dPG‐lPGS (1‐layer) exhibited the least platelet adhesion; however, a significant area of the surface was still occupied by platelets, indicating that a single layer was insufficient to fully prevent adhesion.

In the 5‐layer group, most samples demonstrated improved resistance to platelet adhesion compared to their monolayer counterparts. An exception was PS‐2dPG‐lPGS (5‐layer), which still exhibited noticeable fluorescence, indicating residual platelet attachment. In contrast, PS‐5dPG‐lPGS (5‐layer) and PS‐10dPG‐lPGS (5‐layer) demonstrated significantly cleaner surfaces, with minimal platelet adhesion. The 10‐layer‐coated group showed the best performance overall: across all samples, only a few scattered green spots were observed, indicating effective prevention of platelet adhesion and therefore excellent hemocompatibility.

**Figure 3:**
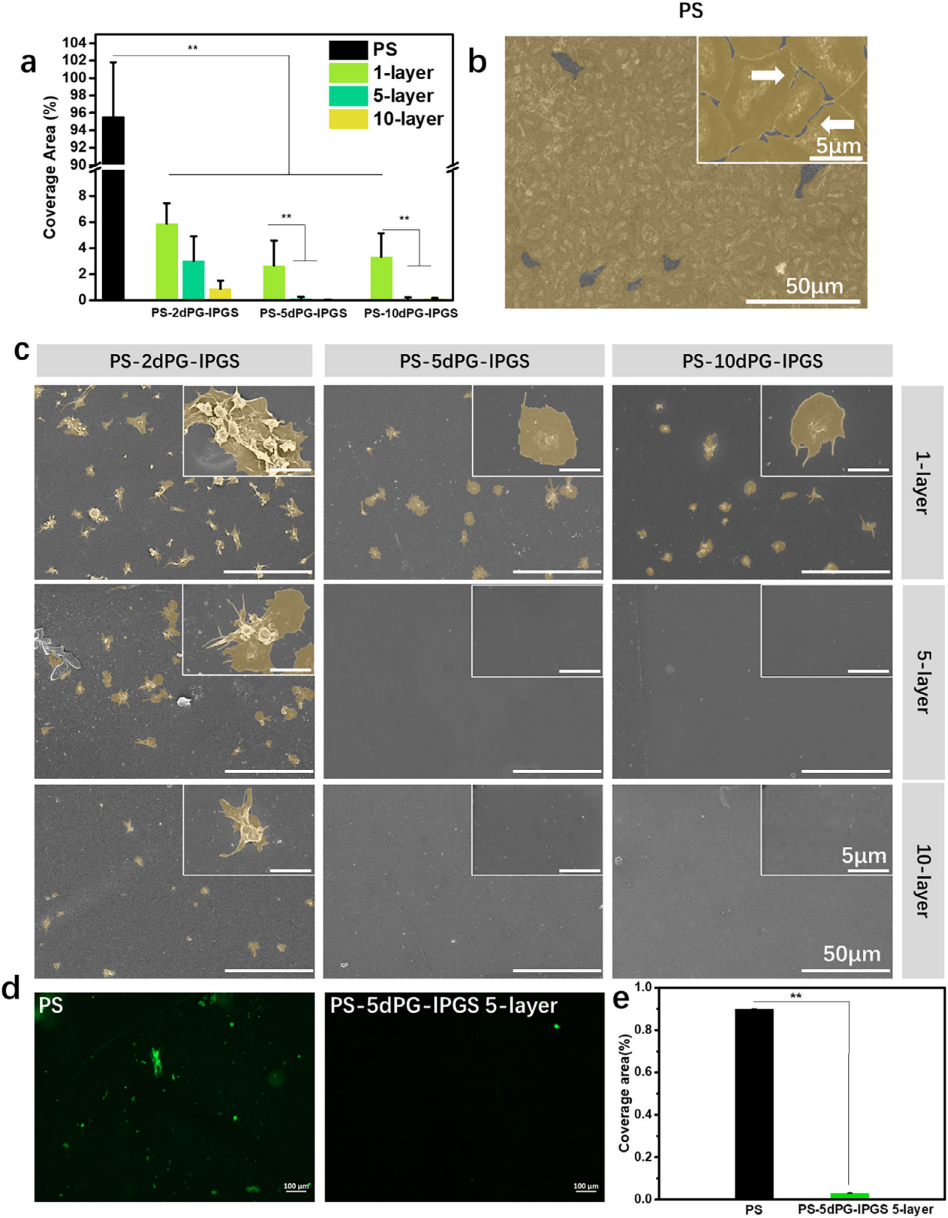
a) Percentage of coverage area by platelets, according to SEM images. b) SEM images of polystyrene surface after platelet incubation at 37 °C for 1 h. c) SEM images of different samples after platelet incubation at 37 °C for 1 h. d) Fluoresence microscopy images of different samples after incubating Fibrinogen from Human Plasma, Alexa Fluor 488 Conjugate at 37 °C for 2 h. e) Percentage of coverage area by fibrinogen, according to fluoresence images ^*^ *p*<0.05, ^**^ *p*<0.01.

To further evaluate platelet activation, the morphology of adhered platelets was examined using SEM (**Figure** **3**b,c). On the unmodified PS surface, extensive platelet coverage was observed, with platelets displaying a fully spread morphology—an indication of strong activation. Their distinct outlines and extended filopodia were clearly visible at 8000 × magnification, highlighting the poor hemocompatibility of the unmodified PS. After surface modification with dPG and lPGS, platelet adhesion was substantially reduced. In the PS‐2dPG‐lPGS monolayer sample, although the number of adhered platelets decreased, some aggregates remained, and platelets retained a highly activated morphology. Similar observations were made for the PS‐5dPG‐lPGS and PS‐10dPG‐lPGS monolayer samples. In contrast, the 5‐layer coatings provided markedly better anti‐adhesive performance. Except for PS‐2dPG‐lPGS (5 layers), which still showed some fully spread platelets, the PS‐5dPG‐lPGS (5 layers) and PS‐10dPG‐lPGS (5 layers) samples exhibited nearly platelet‐free surfaces, confirming significantly improved blood compatibility. This trend continued in the 10‐layer group. PS‐2dPG‐lPGS (10 layers) demonstrated reduced platelet adhesion and lower activation states, while PS‐5dPG‐lPGS and PS‐10dPG‐lPGS (10 layers) presented the cleanest surfaces, with negligible platelet adhesion, verifying their superior hemocompatibility.

In addition, the coverage area of different surfaces was quantitatively analyzed based on more than 40 images. The result was shown in Figure 3a. The unmodified PS surface exhibited a coverage area of 95.58 ± 6.26%, indicating nearly complete surface occupation by platelets. In contrast, the PS‐2dPG‐lPGS group showed a substantial reduction in coverage, with percentages of 5.87 ± 1.57% (1‐layer), 3.01 ± 1.90% (5‐layer), and 0.89 ± 0.61% (10‐layer). Similarly, the PS‐5dPG‐lPGS group exhibited platelet coverage of 2.66 ± 1.93% (1‐layer), 0.11 ± 0.16% (5‐layer), and 0.02 ± 0.03% (10‐layer). For PS‐10dPG‐lPGS, the respective values were 3.33 ± 1.80%, 0.08 ± 0.12%, and 0.10 ± 0.07%. These results confirm that increasing the number of layers and the concentration of the base polymer both contributed to enhanced platelet resistance.

To further assess surface interactions with blood components, lactate dehydrogenase (LDH) assays were conducted to quantify adherent cell debris or damaged platelets (Figure 2d). The unmodified PS surface showed a high optical density (OD) value of 0.57 ± 0.05, while the negative control (bare PS without incubation) was 0.07 ± 0.01. In contrast, all coating groups exhibited significantly reduced OD values. Specifically, for PS‐2dPG‐lPGS, the OD values were 0.17 ± 0.01 (1‐layer), 0.10 ± 0.01 (5‐layer), and 0.09 ± 0.01 (10‐layer). The PS‐5dPG‐lPGS group showed OD values of 0.12 ± 0.02 (1‐layer), 0.10 ± 0.01 (5‐layer), and 0.09 ± 0.01 (10‐layer), while the PS‐10dPG‐lPGS group showed 0.14 ± 0.01, 0.07 ± 0.01, and 0.08 ± 0.01, respectively. These findings support the conclusion that surface modification with polyglycerol‐based coatings significantly reduces platelet adhesion and activation. To better understand the mechanism underlying the anticoagulant effect of the coating, coagulation time assays and protein adsorption measurements were performed. Activated partial thromboplastin time (APTT) measurements (Figure S10, Supporting Information) demonstrated that lPGS significantly prolonged clotting time in a dose‐dependent manner—from 31.90 ± 0.20 s (2 µg mL^−1^) to 41.70 ± 0.60 s (5 µg mL^−1^), 250.30 ± 2.30 s (100 µg mL^−1^), and >500 s (200 µg mL^−1^). Although the APTT prolongation by lPGS was less pronounced than that by heparin (which exceeded 500 s at 100 µg mL^−1^), it still showed substantial anticoagulant activity by effectively interfering with the intrinsic coagulation pathway. Protein adsorption assays (Figure 3d,e) further supported this anticoagulant effect. Fluorescence imaging revealed abundant fibrinogen adsorption on unmodified PS, while the PS‐5dPG‐lPGS 5‐layer coating drastically reduced fibrinogen coverage from 0.90 ± 0.002% to 0.03 ± 0.00005%. Since fibrinogen binding to platelet integrin αIIbβ3 is critical for platelet adhesion and aggregation, the coating's ability to limit fibrinogen adsorption effectively disrupts the platelet activation cascade. Consistently, SEM images showed minimal platelet adhesion on coated surfaces, with platelets exhibiting predominantly round morphology characteristic of a non‐activated state. This observation confirms that the coating suppresses platelet activation and aggregation.

Furthermore, hemolysis assays were conducted according to ISO 10993–4 standards to evaluate red blood cell compatibility (Figure 2b,c). All samples exhibited hemolysis ratios well below 5%, confirming that the modified surfaces are non‐hemolytic and safe for blood‐contacting applications.

In summary, these results demonstrate that the polyglycerol‐based lPGS multilayer coating inhibits thrombus formation through a dual‐action mechanism. First, the highly hydrated and sterically hindered polyglycerol backbone effectively resists nonspecific protein adsorption, as shown by the minimal fibrinogen coverage observed. Second, the densely distributed sulfate groups on lPGS mimic the negatively charged domains of heparin,  facilitating electrostatic interactions with coagulation factors and thereby prolonging the intrinsic coagulation pathway, as evidenced by the significantly extended APTT. Notably, coating performance varied with both the number of layers and the concentration of the base polymer. For instance, the PS‐5dPG‐lPGS coatings with 5 and 10 layers exhibited superior platelet resistance compared to the monolayer, attributed to higher lPGS grafting density and more comprehensive surface coverage by functional macromolecules. Increasing the number of dPG layers introduced additional reactive sites (e.g., –NH_2_ and –CHO), enabling a denser and more uniform lPGS coating. Furthermore, the initial concentration of dPGA‐BP critically influenced the density of the grafted network; for example, within monolayers, PS‐10dPG‐lPGS outperformed PS‐2dPG‐lPGS, likely due to a greater availability of functional groups for subsequent modification. Compared with conventional heparin coatings—which act mainly via antithrombin III‐mediated thrombin inhibition but often suffer from desorption and activity loss under physiological shear^[^ ^19^ ^]^—the covalently assembled lPGS multilayers maintained more stable anticoagulant activity over prolonged exposure. The optimized multilayer matched heparin in anticoagulant and anti‐platelet performance while offering superior protein‐fouling resistance and long‐term stability. Performance was also possibly linked to polymer architecture: longer lPGS chains and higher sulfonation degrees enhanced surface coverage and electrostatic repulsion, reducing protein and platelet adhesion; the branched dPG framework promoted multivalent interactions and uniform growth; and the dual crosslinking strategy (Schiff base and benzophenone photo‐click) improved stability. Optimal crosslinking density is essential, as excessive rigidity may limit chain flexibility and functional group accessibility. Future studies will further explore these structure–function relationships. Considering both preparation efficiency and hemocompatibility, the PS‐5dPG‐lPGS 5‐layer coating was selected for further biological evaluation.

### 2.4 Cytotoxicity

**Figure 4:**
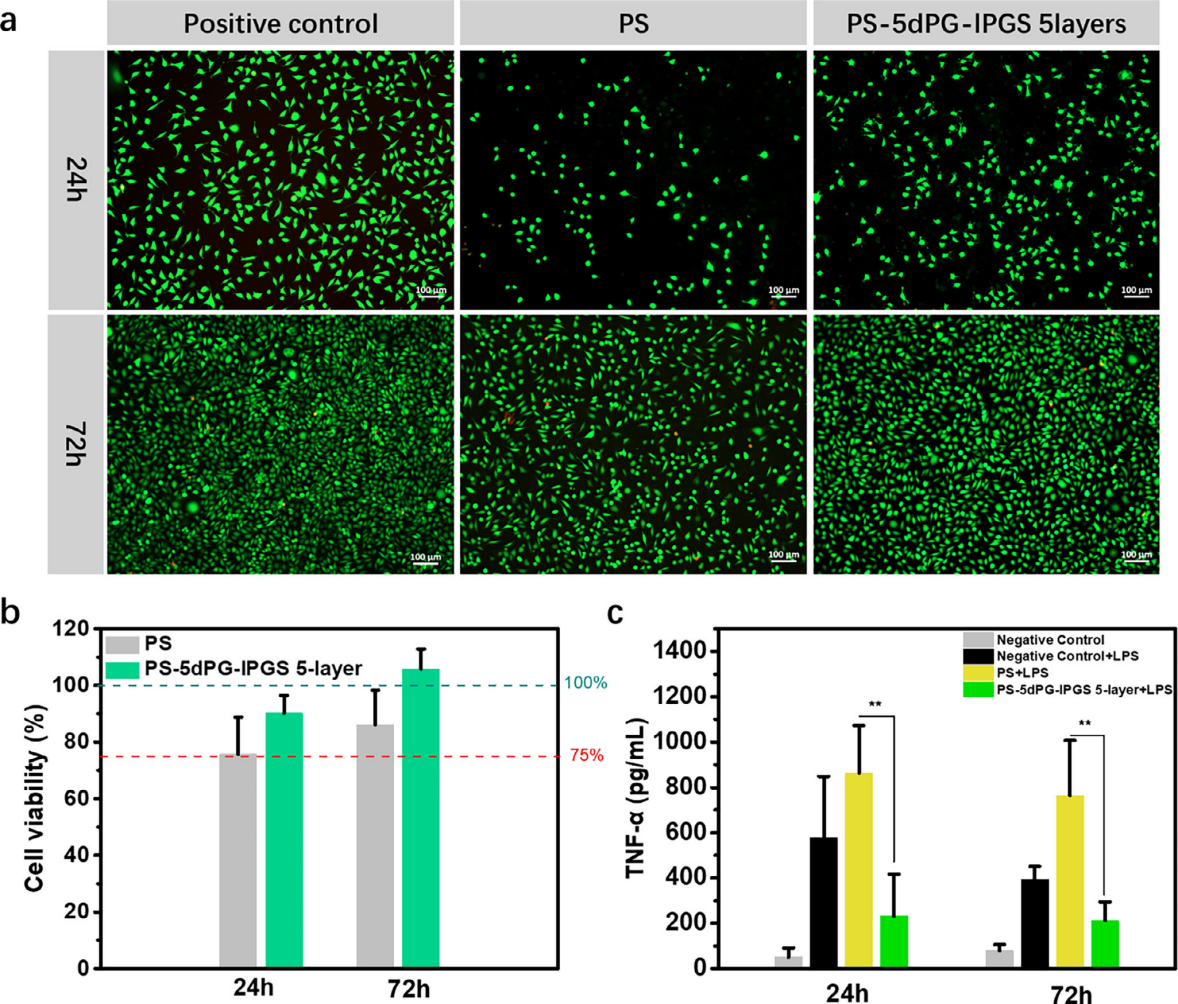
a) Fluorescent images of different samples after incubation with L929 for 24 and 72 h. b) Cell viability of different samples after incubation with L929 for 24 and 72 h. c) TNF‐α concentrations of the medium from different samples incubated with RAW264.7 for 24 and 72 h. ^*^ *p*<0.05, ^**^ *p*<0.01.

Cytotoxicity is a key parameter in evaluating the biocompatibility of biomaterials. In this study, L929 fibroblast cells were chosen to assess the cytotoxicity of the modified surfaces. After incubation, cell viability was analyzed using a live/dead staining assay. As shown in **Figure** **4**a, after 24 h of incubation, the positive control group exhibited the highest cell density with well‐spread morphology, indicative of healthy and adherent cells. In contrast, the unmodified PS surface showed the lowest number of L929 cells, and the cells remained in a spherical shape, indicating their poor adhesion and limited adaptation within this time frame. However, the PS‐5dPG‐lPGS surface showed improved cell behavior. Although cell density was slightly lower than that of the positive control, the cells displayed a healthier morphology with more extensive spreading. After 72 h of co‐incubation, cell density on the PS surface increased compared to the 24‐h time point, but several red‐stained (dead) cells were observed. In contrast, the PS‐5dPG‐lPGS (5‐layer) surface supported robust cell proliferation and spreading, with a morphology and density comparable to the positive control, demonstrating enhanced cytocompatibility. To further quantify cell viability, a CCK‐8 assay was performed (Figure 4b). After 24 h of incubation, the viability of cells on the PS surface was 75.65 ± 13.10%, significantly lower than that observed on the PS‐5dPG‐lPGS (5‐layer) surface (90.21 ± 6.25%). After 72 h, viability on the PS‐5dPG‐lPGS surface increased to 105.88 ± 6.98%, notably higher than the value for on the PS surface (86.10 ± 12.21%). In summary, these results confirm that surface modification with polyglycerol‐based coatings substantially improves the cytocompatibility of PS materials, promoting better cell adhesion, spreading, and proliferation over time.

### 2.5 Preliminary Anti‐Inflammatory Property

Inflammatory responses are a common complication associated with blood‐contacting materials, potentially leading to the failure of implantable medical devices.^[^ ^20^ ^]^ To assess the anti‐inflammatory potential of the developed coating, RAW264.7 macrophages were used as a model system, with lipopolysaccharide (LPS) applied as a pro‐inflammatory stimulus. Tumor necrosis factor‐α (TNF‐α), a representative pro‐inflammatory cytokine, was measured by ELISA after 24 and 72 h of incubation. As shown in Figure 4c, after 24 h of incubation, the concentration of TNF‐α in the negative control group was 51.51 ± 39.94 pg mL^−1^. Upon stimulation with LPS, the concentration increased significantly, to 579.71 ± 268.55 pg mL^−1^, confirming that inflammation was induced successfully. For unmodified PS samples, the TNF‐α levels further increased to 863.17±210.70 pg mL^−1^, suggesting that the PS surface may exacerbate inflammatory response. Conversely, in the PS‐5dPGS‐lPGS (5‐layer) group, TNF‐α concentration significantly decreased, to 231.24 ± 185.34 pg mL^−1^, indicating a pronounced anti‐inflammatory effect. After 72 h of incubation, a similar trend was observed. The TNF‐α concentrations in the negative control, LPS‐treated negative control, LPS‐treated PS, and LPS‐treated PS‐5dPG‐lPGS (5 layers) groups were 80.07 ± 26.03 pg mL^−1^, 394.24 ± 57.41 pg mL^−1^, 764.23 ± 242.93 pg mL^−1^, and 211.93 ± 83.02 pg mL^−1^, respectively. Although the precise mechanism remains to be fully elucidated, the reduced TNF‐α levels observed on the PS‐5dPG‐lPGS 5‐layer surface may be related to the presence of lPGS, which structurally mimics heparin. It is hypothesized that the highly charged sulfated groups may interact with pro‐inflammatory mediators (e.g., LPS or HMGB1), thereby modulating their availability or recognition by macrophage receptors such as Toll‐like receptor 4 (TLR4).^[^ ^21^ ^]^ Further mechanistic studies will be necessary to clarify the underlying interactions and confirm whether these effects of RAW264.7 are directly mediated by the sulfated polyglycerol structure. These results demonstrate that the PS‐5dPG‐lPGS (5‐layer) coating exhibits excellent anti‐inflammatory properties.

### 2.6 Stability and Long‐Term Hemocompatibility Property

To assess coating stability, water contact angle measurements were performed after 1–4 weeks of phosphate‐buffered saline (PBS) immersion under agitation. As shown in Figure S11 (Supporting Information), unmodified PS surfaces showed consistently high contact angles (98.60 ± 1.93°, 92.20 ± 5.85°, 94.73 ± 7.77°, and 98.80 ± 5.06°), reflecting their intrinsic hydrophobicity. In contrast, PS‐5dPG‐lPGS surfaces exhibited significantly lower contact angles (47.60 ± 5.57°, 55.07 ± 5.57°, 51.77 ± 2.91°, and 49.63 ± 4.97°), indicating sustained hydrophilicity. The minimal change in contact angle over time demonstrates that the chemically bonded polyglycerol coating remains stable under physiological conditions, supporting its long‐term applicability for blood‐contacting devices.

**Figure 5:**
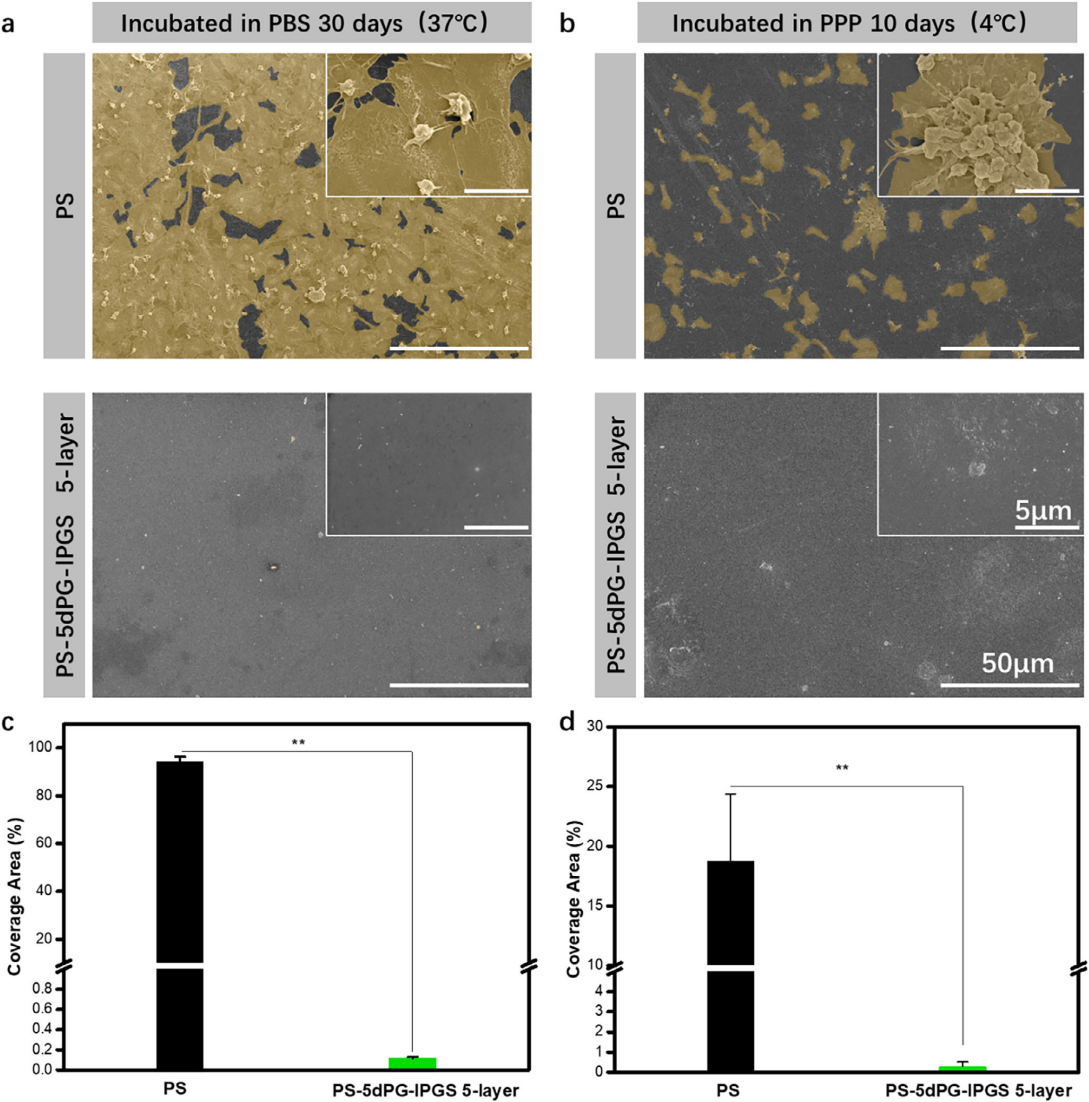
a) SEM images of platelet adhesion results of PS surface and PS‐5dPG‐lPGS 5‐layer surface after 30 days of incubation in PBS. b) SEM images of platelet adhesion results of PS surface and PS‐5dPG‐lPGS 5‐layer surface after 10 days of incubation in PPP. c) Platelet coverage areas of the different surfaces after 30 days incubation in PBS. d) Platelet coverage areas of the different surfaces after 10 days of incubation in PPP. ^*^ *p*<0.05, ^**^ *p*<0.01.

Long‐term hemocompatibility is a crucial property for blood‐contacting materials and implantable devices. In this study, imine bonds connecting different molecules were reduced to more stable amine bonds, allowing polyglycerols to be grafted onto the surface and crosslinked laterally for extended periods. To evaluate the long‐term stability and functionality of the coating, PBS and platelet‐poor plasma (PPP) were used as mimetic liquid environments. After different incubation periods, the materials were re‐evaluated, and the results are shown in **Figure** **5**. For the PBS‐immersed group (Figure 5a), the unmodified PS surface remained covered with highly activated platelets, with a platelet coverage area of 94.35 ± 2.06% (Figure 5c). In contrast, the surface of PS‐5dPG‐lPGS remained clean, with only a few rounds, non‐activated platelets observed. The platelet coverage area was significantly lower at 0.12 ± 0.01%. Even after 30 days of immersion in PBS, the coating maintained excellent anti‐coagulation properties. This robust performance can be attributed to the chemical stability of the polymer network provided by the covalent amine bonds formed during the reduction step. These results demonstrate that the coating remained stable and functional in PBS over extended periods.

To further assess the coating's performance under more biologically relevant conditions, platelet‐poor plasma, which contains a variety of proteins and biomolecules, was used. After incubation, a short‐term static platelet adhesion assay was conducted using fresh platelet‐rich plasma from the same donor and blood batch to ensure platelet functionality and reduce variability. The samples were immersed for 10 days before re‐evaluation. On the PS surface (Figure 5b), platelet adhesion and aggregation were still observed, with a platelet coverage area of 18.76 ± 5.61% (Figure 5d). Although this value was lower than that observed in the PBS group, it does not indicate improved hemocompatibility. The apparent reduction in platelet coverage likely resulted from platelet degradation during the extended incubation outside the human body, leading to diminished platelet viability and function. Despite the overall reduction in platelet activity, the PS surface still induced platelet activation, underscoring the need for surface modification. In contrast, the PS‐5dPG‐lPGS surface remained largely free of adhered platelets, with a coverage area of only 0.28 ± 0.23%. These findings demonstrate that the polyglycerol coating remains stable and functional over time, even in complex biological environments. It should be noted that these long‐term hemocompatibility assessments were performed under static in vitro conditions using PBS and PPP, which do not fully replicate the dynamic blood flow and continuous renewal of proteins and cells encountered in vivo. Therefore, while the coating exhibited durable anti‐adhesive properties and stability over extended incubation, these results indicate prolonged in vitro hemocompatibility rather than definitive long‐term anticoagulant efficacy. Further validation using dynamic flow systems and in vivo models is necessary to confirm the coating's long‐term hemocompatibility performance.

In conclusion, the PS‐5dPG‐lPGS coating exhibits excellent long‐term stability and antiplatelet performance in both PBS and PPP environments. This suggests its potential for significantly extending the functional lifespan of blood‐contacting materials.

## 3 Conclusion

In this study, a polyglycerol‐based heparin‐mimetic coating was successfully grafted onto polystyrene surfaces using an LBL assembly method, significantly improving its physicochemical and biological properties. Surface characterization—including AFM imaging, elemental analysis, and ellipsometry—confirmed uniform polymer grafting with increased coating thickness and enhanced surface roughness. TBO dye‐binding assays quantitatively verified a high surface density of sulfonic acid groups, correlating with improved negative charge density. Wettability measurements showed a significant reduction in water contact angle, indicating improved surface compatibility. Cytotoxicity assays confirmed the biocompatibility of the coating, as L929 fibroblast cells adhered and proliferated well on the modified surfaces. The modified surfaces exhibited excellent hemocompatibility by reducing protein adsorption, platelet adhesion and activation, as demonstrated by fluorescence imaging and SEM analysis. The multilayer coatings showed highly effective resistance to platelet adhesion. Additionally, preliminary anti‐inflammatory tests using RAW264.7 macrophages demonstrated that the polyglycerol coating effectively suppressed TNF‐α secretion, suggesting reduced immune response. Long‐term stability tests in PBS and PPP further confirmed that the coating remained functional over time, preventing platelet adhesion even after extended incubation. Overall, the polyglycerol‐based coating effectively enhanced the hemocompatibility of PS surfaces, making it a promising candidate for blood‐contacting biomedical applications such as implants and medical devices.

## 4 Experimental Section

### Materials

All chemicals and solvents were used as received and purchased from Thermo Fisher Scientific. Human blood for research purposes was obtained from the German Red Cross (DRK‐Blutspendedienst Nord‐Ost). Ultrapure water (18.2 MΩ·cm, Milli‐Q system, Millipore, USA) was used in all experiments.

### Synthesis—Preparation of EEGE

EEGE was synthesized from 2,3‐epoxypropan‐1‐ol(glycidol) and ethylvinyl ether according to previous method.^[^ ^22^ ^]^

### Synthesis of Linear Polyglycerol‐Block‐Poly (allyl glycidyl ether) (lPG‐b‐AGE)

A total of 0.137 g (1 equiv) of tetra‐n‐octylammonium bromide was weighed into a flask and heated at 120 °C under vacuum for 2 h to remove residual water and oxygen. Subsequently, 10 mL of extra‐dry toluene and 5 mL (137 equiv) of EEGE were added to the flask. Triisobutylaluminum (0.673 mL) was then introduced as a catalyst, and the reaction was allowed to proceed for 2 h. After this, 0.595 mL (20 equiv) of AGE was injected, followed by another 0.673 mL of triisobutylaluminum. The mixture was stirred overnight to ensure complete reaction. To terminate the polymerization, 1 mL of methanol was added. The reaction mixture was concentrated, and impurities were precipitated using diethyl ether. After centrifugation, the product was re‐concentrated and dissolved in tetrahydrofuran (THF). To isolate lPG‐b‐AGE, hydrochloric acid (HCl) was added dropwise to induce precipitation. The resulting solid was redissolved in methanol and subjected to dialysis for three days. Finally, the purified product was stored in methanol in the freezer (‐20 °C).

### Preparation of lPGS

A total of 500 mg (1 equiv) of IPG‐b‐AGE was fully dissolved in 30 mL of dimethylformamide (DMF). In a separate container, 3.27 g (411 equiv) of sulfur trioxide–pyridine complex was dissolved in 20 mL of DMF. The sulfur trioxide–pyridine solution was added dropwise to the polymer solution under stirring, and the reaction was allowed to proceed at room temperature for 24 h. After completion, the product was purified by dialysis and collected by lyophilization. To introduce amine functionality onto the surface, the obtained polyglycerol sulfate was further modified via a thiol–ene click reaction. Specifically, 1 g (1 equiv) of polyglycerol sulfate, 385 mg (50 equiv) of cysteamine, and 50 mg of the photoinitiator Irgacure 2959 were dissolved in 50 mL of deionized water. To remove dissolved oxygen, nitrogen was bubbled through the solution for 20 min. The reaction mixture was then irradiated with 370 nm UV light for 1.5 h. Following irradiation, the product was dialyzed against deionized water for 3 days and subsequently lyophilized to obtain the final product, referred to as lPGS.

### Preparation of dPGA and dPG‐CHO

dPGA was synthesized according to a previously reported method.^[^ ^23^ ^]^ For the synthesis of dPG‐CHO, 1 g (1 equiv) of dPG was fully dissolved in 30 mL of distilled water. Separately, 0.95 g (44 equiv) of sodium periodate was dissolved in 10 mL of distilled water and added dropwise to the dPG solution under constant stirring. The oxidation reaction was conducted in the dark for 12 h to prevent degradation of the reactants. Following the reaction, the mixture was purified by dialysis against distilled water for 3 days, and the final product was obtained via lyophilization.

### Preparation of Benzophenone Grafted dPGA

A total of 500 mg (1 equiv) of dPGA was dissolved in 35 mL of distilled water in a round‐bottom flask. Separately, 226.2 mg (20 equiv) of 4‐benzoylbenzoic acid, 288 mg (50 equiv) of ethyl‐3‐(3‐dimethylaminopropyl) carbodiimide (EDC), 287.7 mg (50 equiv) of N‐hydroxysuccinimide (NHS), and 183.7 µL (50 equiv) of triethylamine were dissolved in 10 mL of DMF and allowed to activate for 1 h at room temperature. The activated ester solution was then added dropwise to the aqueous dPGA solution under constant stirring. The reaction was carried out in the dark for 12 h to prevent photodegradation. After completion, the mixture was purified by dialysis against distilled water for 3 days, and the final product, dPGA‐BP, was obtained via lyophilization.

### Coating Preparation

To initiate surface functionalization, dPGA‐BP was dissolved in distilled water at concentrations of 2, 5, and 10 mg mL^−1^. Polystyrene (PS) sheets were immersed in these solutions for 1 h under dark conditions to allow for adsorption. After immersion, the PS sheets were dried and subsequently exposed to ultraviolet (UV) irradiation (PR160‐370 nm Gen 2, Kessil, USA) for 20 min to induce covalent photo‐crosslinking between the benzophenone moieties and the PS surface. Following UV treatment, the modified PS surfaces were subjected to an LBL assembly process by alternating immersion in dPGA (2 mg mL^−1^) and dPG‐CHO (6 mg mL^−1^) solutions for varying durations, enabling the formation of imine‐linked multilayers. To functionalize the outermost layer, the samples were immersed in lPGS solution (10 mg mL^−1^), allowing for the grafting of polyglycerol sulfate. Finally, the samples were treated with NaBH_4_ to reduce imine bonds to more stable secondary amines, thereby enhancing the structural stability of the multilayer coating.

### Physical and Chemical Characterization—Characterization of Polymers

The block copolymer and the functionalized polyglycerol derivatives were characterized using ^1^H NMR, GPC, and FTIR. ^1^H NMR spectra were recorded on a Jeol Eclipse 500 MHz spectrometer (Tokyo, Japan) to confirm chemical structures and determine the degree of functionalization. Molecular weights and polydispersity indices were analyzed using GPC (Agilent Technologies, Santa Clara, CA, USA). FTIR spectra were collected on a PerkinElmer instrument (USA) to identify characteristic functional groups. Detailed experimental procedures and analysis conditions are provided in the Supporting Information.

### Characterization of Coatings

The surface morphology of both unmodified and coated samples was examined using SEM with a Hitachi SU8030 (Japan) operated at an accelerating voltage of 5 kV. Elemental composition and chemical states of surface elements were analyzed by XPS using an Axis Ultra DLD spectrometer (Manchester, UK). Surface hydrophilicity was assessed via static water contact angle measurements using a goniometer system from DataPhysics Instruments (Germany). The grafting density of ‐SO_3_^−^ was measured by Toluidine Blue O dying assay. The absorbance at 633 nm was measured using a microplate reader (Tecan SPARK, Switzerland). The thickness of the coatings was determined by ellipsometry using a Sentech SenPro system (Germany). The roughness of the surfaces was measured by JPK‐nanowizzard(Germany). Detailed protocols for each characterization method are provided in the Supporting Information.

### Hemocompatibility—Static Platelets Adhesion and Activation

Whole human blood was centrifuged at 1500 rpm for 15 min to obtain PRP. Samples (5 × 6 mm) were immersed in PRP and incubated at 37 °C for 45 min. After incubation, the PRP was carefully removed, and the samples were rinsed three times with PBS to eliminate non‐adherent platelets. Some of the samples were stained with Calcein AM and Ethidium Homodimer‐1 (EthD‐1), rinsed again with PBS, and then observed under a fluorescence microscope (ZEISS Axio Observer.Z1, Germany) to visualize adherent live and dead platelets. The remaining samples were then fixed in 2.5% glutaraldehyde solution overnight at 4 °C. Following fixation, the samples underwent gradient ethanol dehydration and were sputter‐coated with a conductive layer using an Emscope SC 500 sputter coater (Quorum Technologies, UK). Platelet adhesion and activation were subsequently analyzed using SEM.

### Lactate Dehydrogenase Test

Samples (10 × 10 mm) were co‐incubated with PRP at 37 °C. After incubation, the samples were rinsed three times with PBS to remove unbound platelets. The samples were then immersed in 400 µL of lysis solution for 1 h to lyse the platelets. Following lysis, 100 µL aliquots of the supernatant were transferred to a 96‐well plate. To each well, 50 µL of working solution was added, and the plate was incubated for an additional hour. After incubation, 50 µL of stop solution was added to each well. The absorbance at 490 nm was measured using a microplate reader to quantify platelet activation.

### Hemolysis

Whole blood was centrifuged at 1500 rpm for 15 min to isolate erythrocytes. The erythrocyte fraction was collected and resuspended in PBS to prepare a 4% erythrocyte suspension. Samples (5 mm × 5 mm) were immersed in the suspension within 1.5 mL Eppendorf tubes and incubated at 37 °C for 2 h. After incubation, the samples were centrifuged at 3000 rpm for 3 min, and 100 µL of the supernatant was transferred to a 96‐well plate. The absorbance at 540 nm was measured using a microplate reader to assess hemolysis.

### Fibrinogen Adsorption Assay

Fibrinogen from human plasma, conjugated with Alexa Fluor 488, was dissolved in PBS to a final concentration of 0.5 mg mL^−1^. Sample substrates (5 mm × 6 mm) were placed in a 48‐well plate and immersed in the fibrinogen solution, followed by incubation at 37 °C for 2 h. After incubation, the samples were rinsed three times with PBS to remove unbound fibrinogen. The adsorbed fluorescent fibrinogen was then visualized and imaged using a fluorescence microscope.

### APTT Assay

APTT was measured using a C.K. PREST Kit according to the manufacturer's instructions. Lyophilized control plasma was reconstituted, and reagents were equilibrated at room temperature for 30 min. In a preheated Start Max device, 50 µL plasma, 50 µL activator, a magnetic stirrer, and 2 µL sample were incubated at 37 °C for 3 min. Clotting was initiated by adding 50 µL preheated CaCl_2_, and the time to clot formation was recorded. Tests were performed in triplicate with deionized water and heparin as negative and positive controls.

### Cytotoxicity

Murine fibroblast L929 cells were obtained from the German Collection of Microorganisms and Cell Cultures (Braunschweig, Germany) and cultured in Dulbecco's Modified Eagle Medium (DMEM; high glucose, GlutaMAX, Gibco) supplemented with 10% (v/v) fetal bovine serum (FBS, Gibco) and 1% (v/v) penicillin‐streptomycin (Gibco). Samples (10 mm × 8 mm) were placed in a 24‐well plate, and L929 cells were seeded at a density of 2 × 10⁴ cells/well. The cells were cultured for 24 and 72 h. After incubation, the culture medium was replaced with fresh medium containing the CCK‐8 reagent and incubated for an additional 3 h. Subsequently, 150 µL of the supernatant from each well was transferred to a 96‐well plate, and absorbance at 450 nm was measured using a microplate reader to assess cell viability. Live‐dead staining was performed to further evaluate cytotoxicity. Cells were incubated with a staining solution containing 2 µM calcein‐AM and 4 µM ethidium homodimer‐1 (200 µL per well) at 37 °C for 30 min. After three washes with PBS, fluorescent images were acquired using a Zeiss Axio Observer Z1 microscope.

### Preliminary Anti‐Inflammatory Evaluation

The anti‐inflammatory response was evaluated using RAW 264.7 macrophages, which were obtained from the same supplier as the L929 cells and cultured under identical conditions. Samples (10 mm × 8 mm) were placed in a 24‐well plate, and RAW 264.7 cells were seeded at a density of 4 × 10⁴ cells mL^−1^ onto the surface of the samples. The cells were cultured for 48 h, after which the medium was replaced with fresh DMEM containing 1 ng mL^−1^ LPS and incubated for an additional 12 h. After this incubation, the LPS‐containing medium was replaced with standard DMEM, and culture supernatants were collected at 24 and 72 h for TNF‐α quantification using a mouse ELISA assay kit, according to the manufacturer's instructions.

### Stability

PS and PS‐5dPG‐lPGS 5‐layer samples were immersed in PBS for 1, 2, 3, and 4 weeks under shaking condition. At each time point, the samples were dried, and their surface wettability was evaluated by measuring the water contact angle.

### Long‐Term Hemocompatibility

Samples were immersed in PBS under static conditions at 37 °C for 30 days to assess their long‐term hemocompatibility. After incubation, the samples were rinsed and analyzed for platelet adhesion using SEM. To further evaluate hemocompatibility, human blood was centrifuged at 3000 rpm for 15 min to isolate PPP. The samples were then incubated with PPP under static conditions, containing 1% penicillin‐streptomycin, at 4 °C for 10 days. Following incubation, the samples were washed three times with PBS and then incubated with PRP for 45 min. After additional PBS washes, platelet adhesion was again evaluated by SEM.

### Statistical Analysis

More than three parallel samples were conducted for each assay to ensure statistical reliability. Statistical analysis was performed using SPSS software, where a one‐way analysis of variance (ANOVA) test was applied to compare the experimental results. All quantitative data are presented as mean ± standard deviation (SD). Statistical significance was considered for p‐values less than or equal to 0.05.

## Conflict of Interest

The authors declare no conflict of interest.

## Supporting information



Supporting Information

## Data Availability

The data that support the findings of this study are available from the corresponding author upon reasonable request.
