# Supplementary material for: A Robust Heparin‐Mimicking Polyglycerol‐Based Coating for Blood‐Contacting Devices with Long‐Term Hemocompatibility and Preliminary Anti‐Inflammatory Properties
Source: Adv Healthc Mater. 2025 Sep 21;15(2):e02766. doi: 10.1002/adhm.202502766 (PMC12805611; doi:10.1002/adhm.202502766)
Supplement: Supplementary file 1 — Supporting Information [file ADHM-15-0-s001.docx]

A robust heparin-mimicking polyglycerol-based coating for blood-contacting devices with long-term hemocompatibility and preliminary anti-inflammatory properties

Kunpeng Liu^a^, Philip Nickl^a^, Jun Feng^a^*^&^, and Rainer Haag^a^*

1. Institute for Chemistry and Biochemistry, Freie Universität Berlin, Takustr. 3, 14195 Berlin, Germany

*Email: [jun.feng@fu-berlin.de](mailto:jun.feng@fu-berlin.de) and [haag@chemie.fu-berlin.de](mailto:haag@chemie.fu-berlin.de)

&. Current email and address: [jun.feng@leibniz-inm.de](mailto:jun.feng@leibniz-inm.de), INM-Leibniz Institute for New Materials, Campus D2 2, 66123 Saarbrücken, Germany

*Email: [jun.feng@fu-berlin.de](mailto:jun.feng@fu-berlin.de) and [haag@chemie.fu-berlin.de](mailto:haag@chemie.fu-berlin.de)

**Experiment section.**

**Characterization of Polymers**

All ¹H NMR spectra were recorded at 300 K on a JEOL Eclipse 500 MHz spectrometer. Chemical shifts (δ) are reported in parts per million (ppm), referenced to the residual solvent peak as an internal standard. Gel permeation chromatography (GPC) analyses were performed in aqueous solution using an Agilent 1100 system equipped with an automatic injector, isocratic pump, and differential refractive index detector. Fourier-transform infrared (FTIR) spectra were acquired using a Spectrum Two™ FT-IR spectrometer.

**Characterization of Coatings**

**X-ray Photoelectron Spectroscopy:** XPS experiments were performed with an EnviroESCA spectrometer (SPECS Surface Nano Analysis GmbH, Berlin, Germany), equipped with a monochromatic Al Kα X-ray source (Excitation Energy = 1486.71 eV) and a PHOIBOS 150 electron energy. XP spectra were measured in normal emission, and a source-to-analyzer angle of 55° was used. All spectra were acquired in fixed analyzer transmission (FAT) mode. The binding energy scale of the instrument was calibrated, following a technical procedure provided by SPECS Surface Nano Analysis GmbH (calibration was performed according to ISO 15472). For quantification, the survey spectra were acquired at ultra-high vacuum conditions (p < 1 x 10^-5^ mbar) with a pass energy of 100 eV, and the spectra were quantified utilizing the empirical sensitivity factors that were provided by SPECS Surface Nano Analysis GmbH (the sensitivity factors were corrected with the transmission function of the spectrometer). For charge compensation, the highly-resolved XP spectra were acquired under near-ambient pressure conditions (p_H2O_ = 5 mbar) with a pass energy of 50 eV, and the respective data were fitted using UNIFIT 2020 data processing software. For fitting, a Shirley background and a Gaussian/Lorentzian sum function [peak shape model GL (30)] were used. If not denoted otherwise, the L-G mixing component was set to 0.30 for all carbon peaks and 0.40 for all heteroatom peaks. All binding energies were calibrated to the signal observed for the aliphatic C–C bond component (*E_bind_* = 285 eV) if not stated otherwise.

**Scanning Electron Microscopy (SEM):** Samples were cut into 5 mm × 4 mm pieces, rinsed with distilled water, and thoroughly dried. A thin layer of gold was sputter-coated onto the sample surfaces prior to imaging by SEM.

**AFM test:** A 2 wt% polystyrene (PS) solution in toluene was prepared, and 50 μL was deposited onto Si/SiO₂ wafers (10 mm × 10 mm), followed by spin coating. After solvent evaporation, the resulting PS layer was rinsed. Different surface modifications were then applied to the PS layer. The surface topography of each sample was analyzed using a JPK NanoWizard atomic force microscope.

**Toluidine blue O dying-binding assay:** Samples were cut into 5 mm × 6 mm pieces, rinsed with distilled water, and dried. According to the previous research, this test was performed.^[1]^Each sample was immersed in a TBO solution (5 mM in deionized water) with pH adjusted to 10 using 1 M NaOH. After incubation for 6 h, the samples were washed three times with 1 mM NaOH solution. The bound dye was then released by immersing each sample in 50% (v/v) acetic acid solution under mechanical shaking for 24 h. All procedures were conducted in the dark. The absorbance of the resulting solutions was measured at 633 nm using a microplate reader.

**Water Contact Angle Measurement:** Samples were prepared by cutting into 5 mm × 4 mm dimensions, rinsed with distilled water, and dried. Static water contact angles were measured using the sessile drop method. Mean values were calculated from multiple measurements for each sample.

**Thickness Measurement:** A 2 wt% PS solution in toluene was prepared, and 50 μL was deposited onto Si/SiO₂ wafers (11 mm × 11 mm), followed by spin coating. After solvent evaporation, the resulting PS layer was rinsed, and its thickness was measured. Subsequent coatings were deposited onto the PS surface, and their thicknesses were determined using an ellipsometer.

**Supporting Figures**.

Figure S1. The polymerization and functionalization of block polymer.


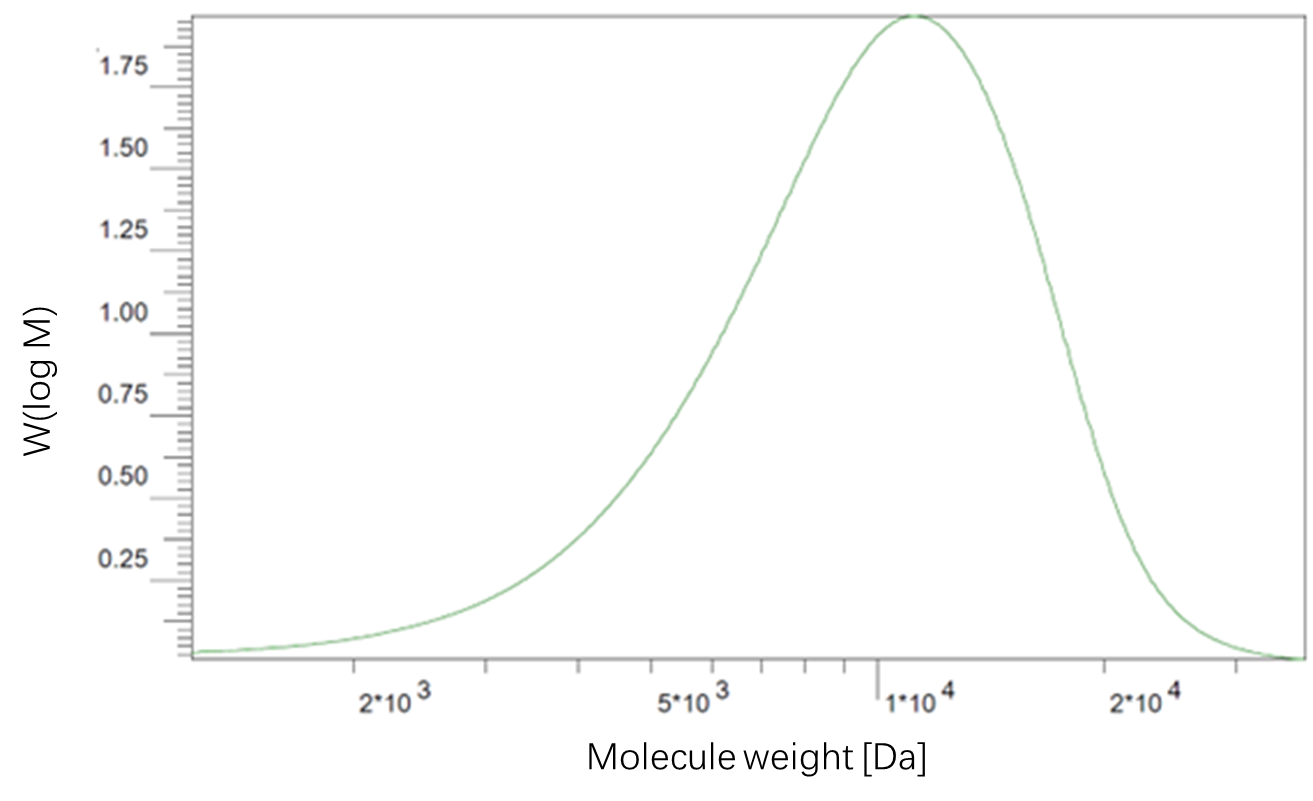


Figure S2. The result of GPC of lPG-b-AGE.


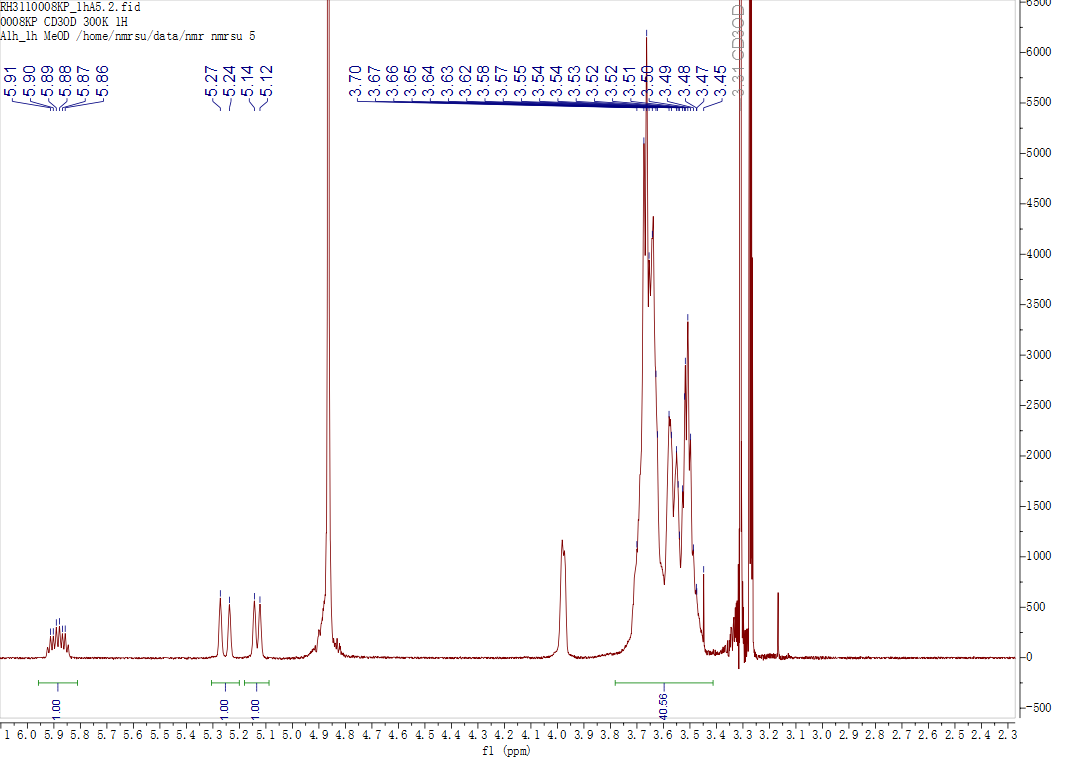


Figure S3. The ^1^H NMR result of lPG-b-AGE in Deuterated Methanol


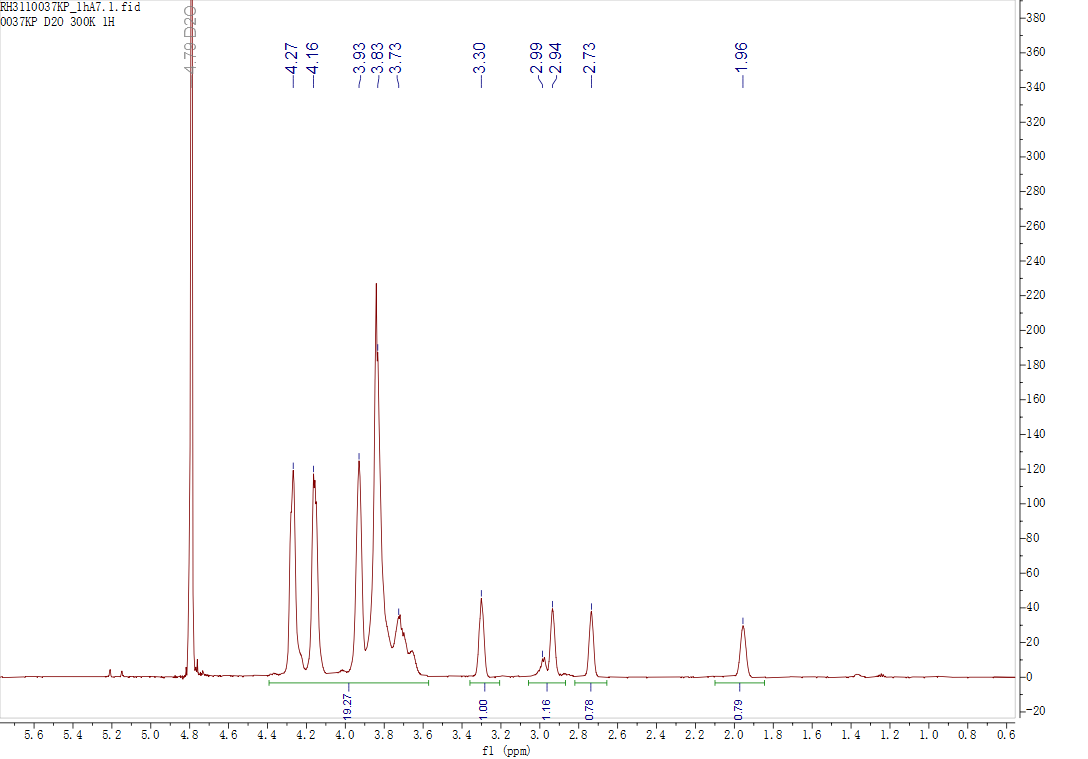


Figure S4. The ^1^H NMR result of lPGS in Deuterium Oxide


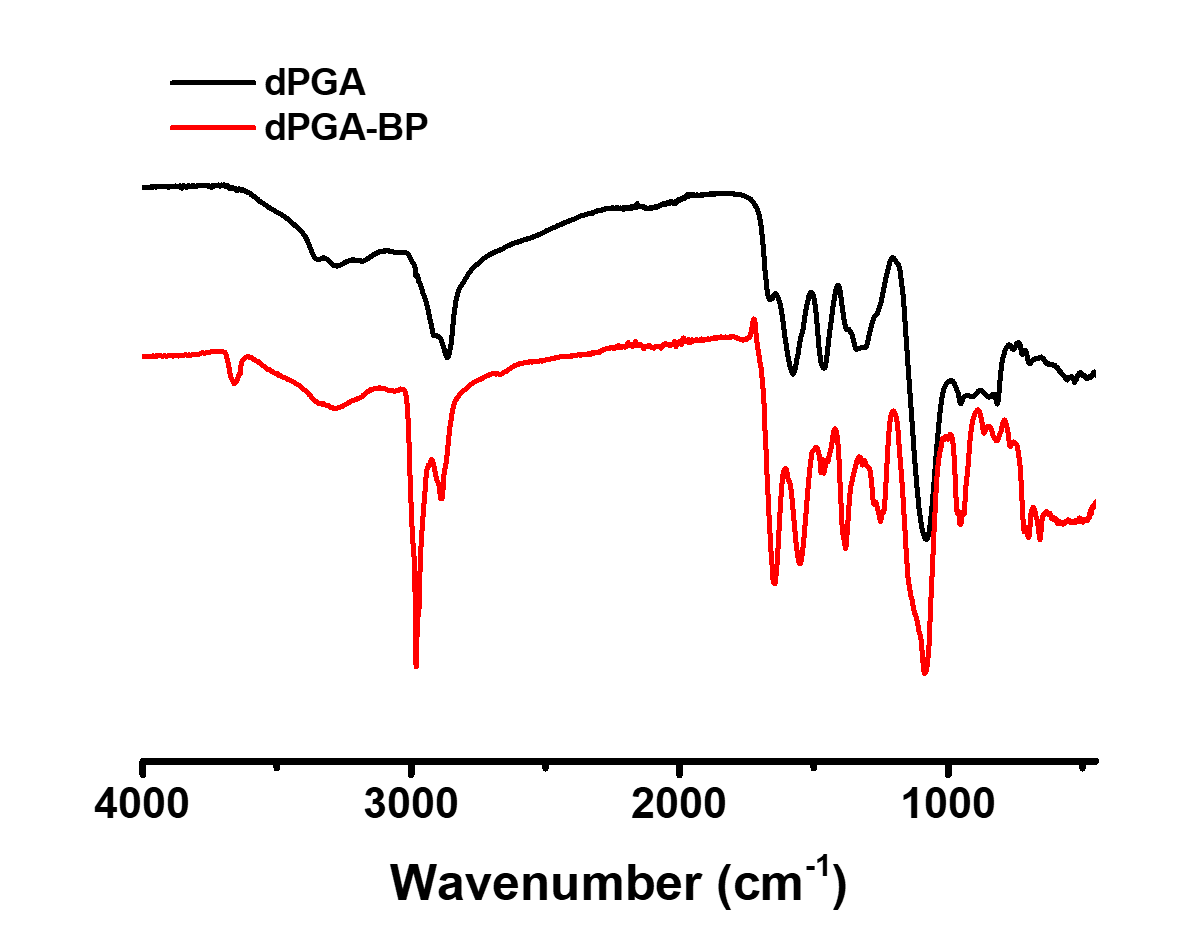


Figure S5. The FTIR result of dPGA and dPGA-BP


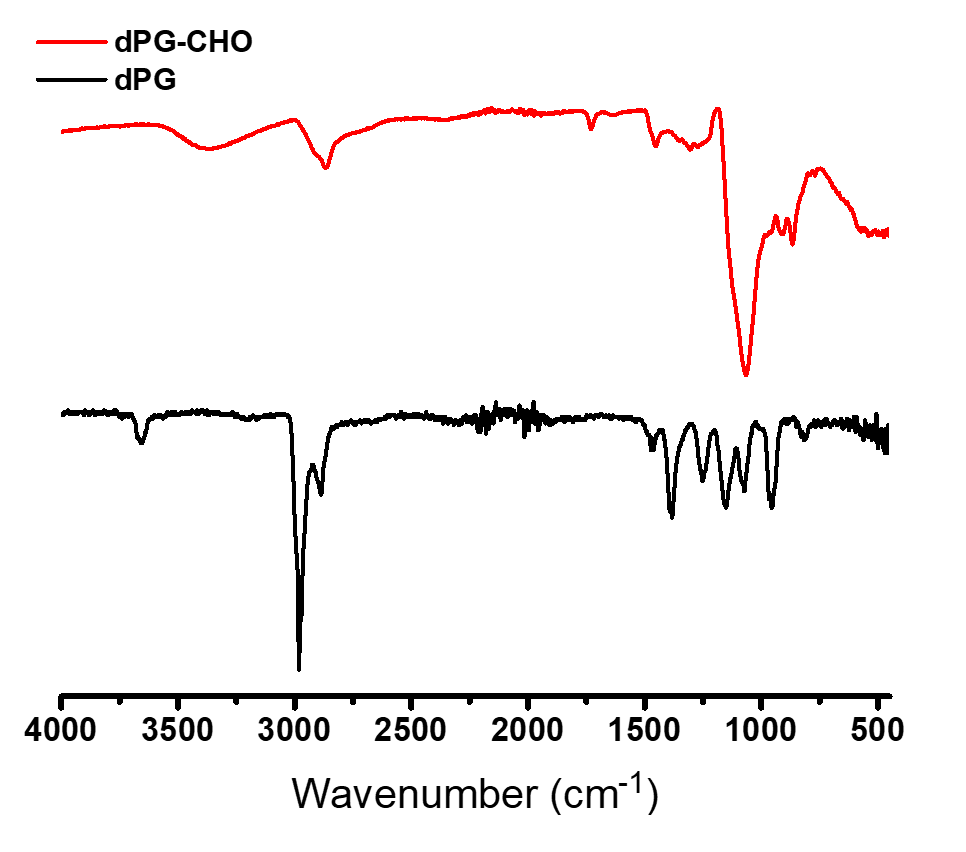


Figure S6. The FTIR result of dPG and dPG-CHO.


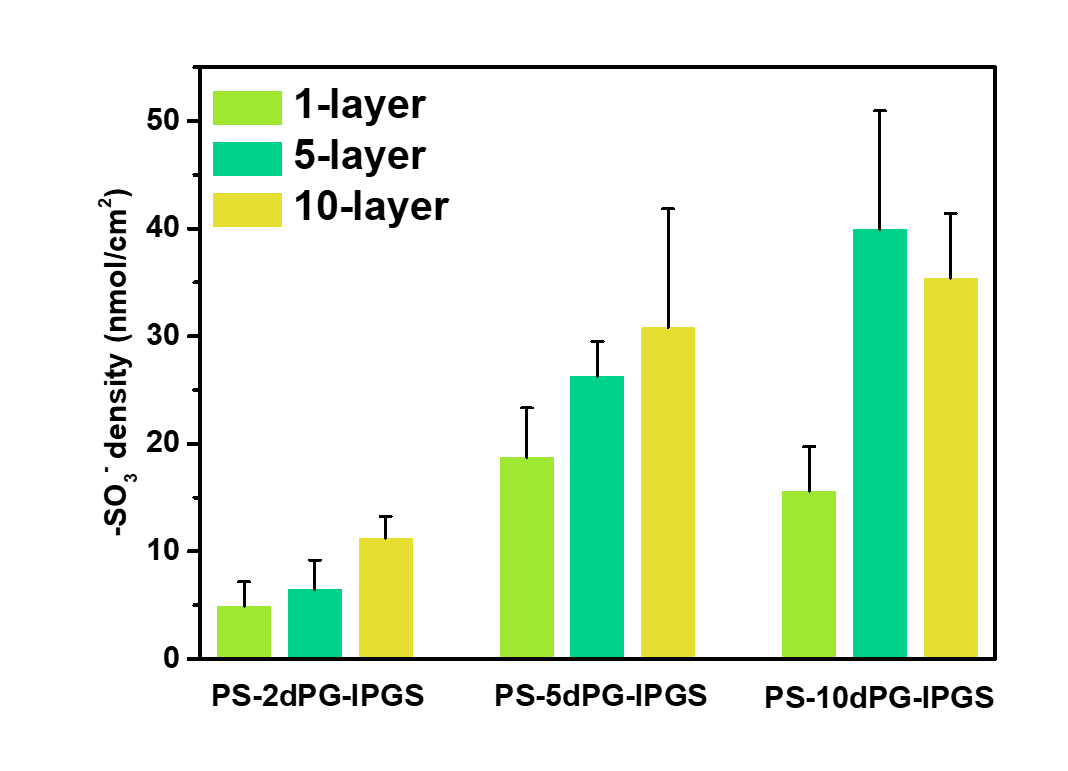


Figure S7. The -SO_3_^-^ density of the different sample surfaces.


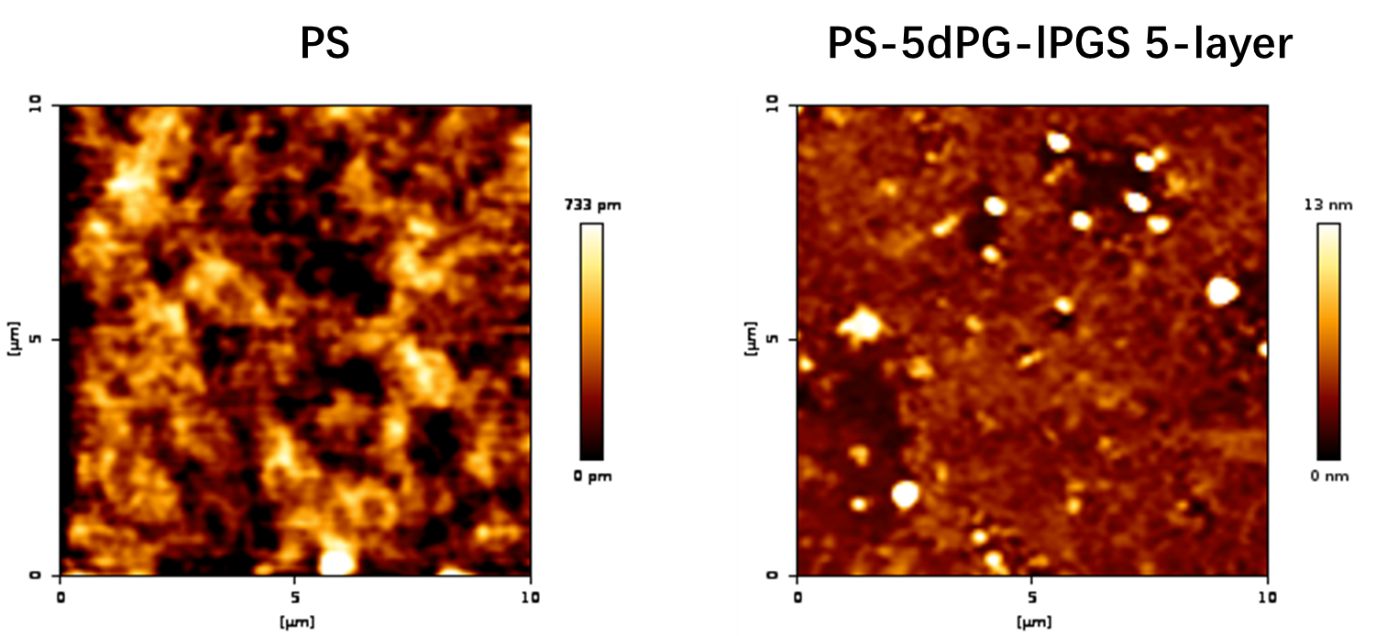


Figure S8. The surface morphology of PS and PS-5dPG-lPGS 5-layer measured by AFM.


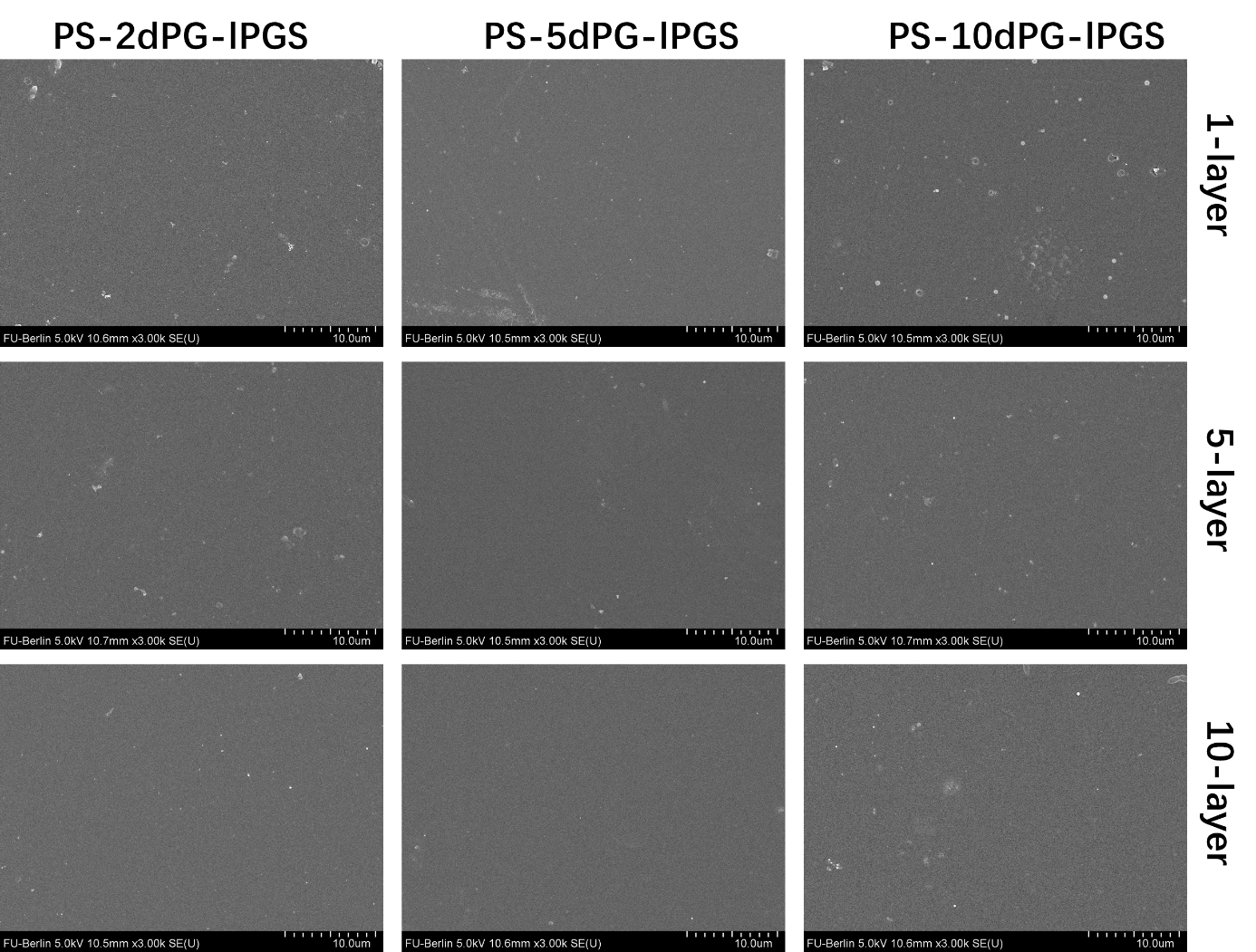


Figure S9. The morphology of samples measured by SEM.


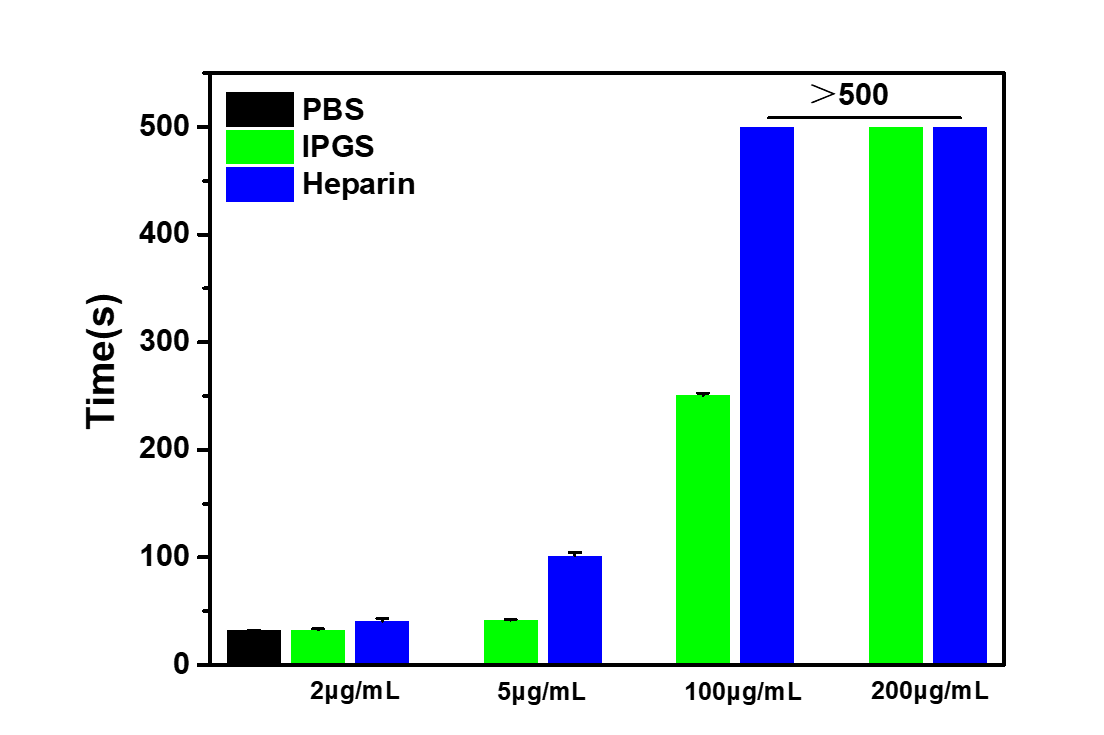


Figure S10. The APTT results of PBS, lPGS and heparin.


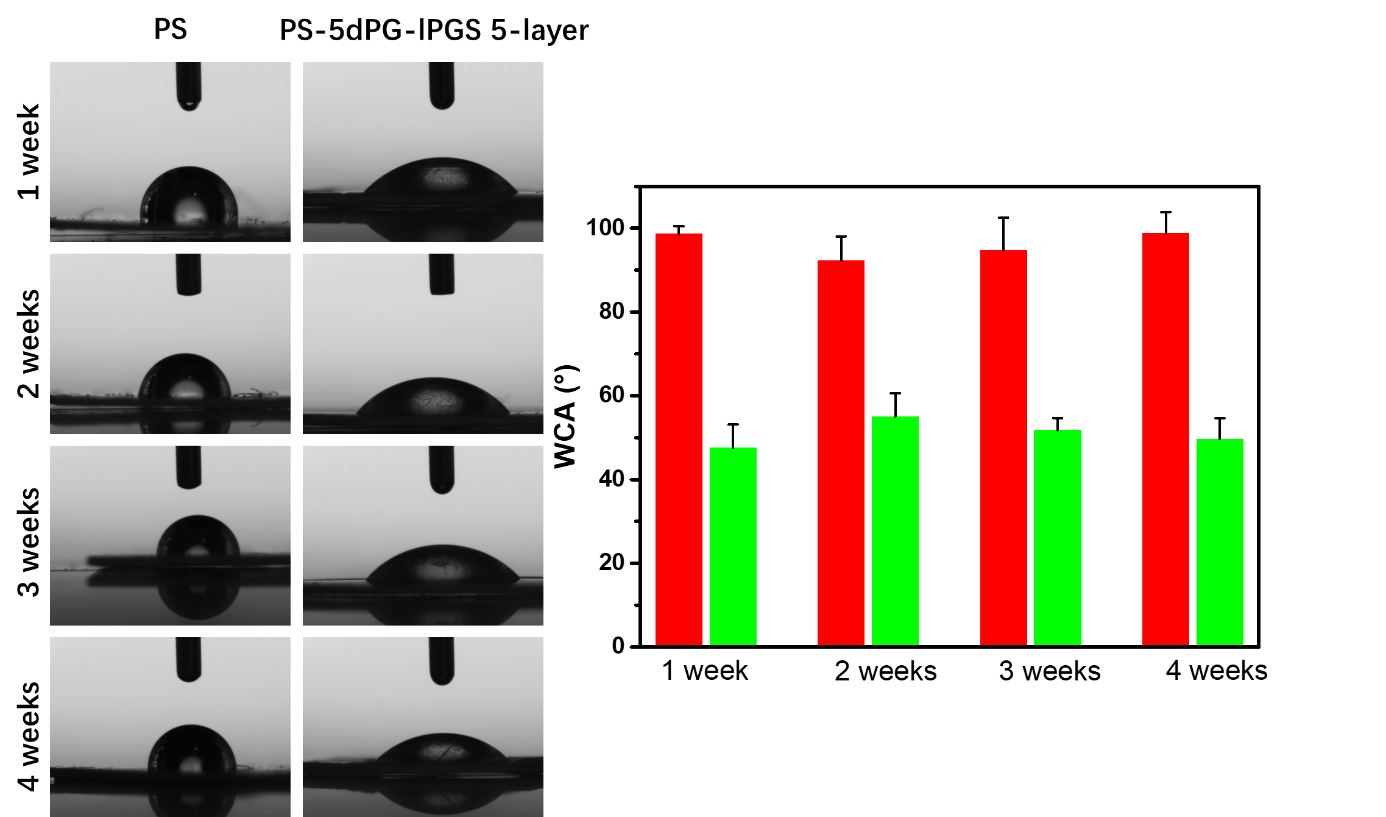


Figure S11. The water contact angle of PS and PS-5dPG-lPGS 5-layer after incubation with PBS for different time periods. For the column bar, red columns represent PS, green columns represent PS-5dPG-lPGS 5-layer.

[1] A. Rangel, T. N. Nguyen, V. Migonney, *J Appl Spectrosc+* **2022**, 89, 552.
